# Acute glucose fluctuation impacts microglial activity, leading to inflammatory activation or self-degradation

**DOI:** 10.1038/s41598-018-37215-0

**Published:** 2019-01-29

**Authors:** Cheng-Fang Hsieh, Ching-Kuan Liu, Ching-Tien Lee, Liang-En Yu, Jiz-Yuh Wang

**Affiliations:** 1 Department of Neurology, Kaohsiung Medical University Hospital, Kaohsiung Medical University, Kaohsiung, Taiwan; 2 Graduate Institute of Medicine, College of Medicine, Kaohsiung Medical University, Kaohsiung, Taiwan; 3 Division of Geriatrics and Gerontology, Department of Internal Medicine, Kaohsiung Medical University Hospital, Kaohsiung Medical University, Kaohsiung, Taiwan; 4 Department of Nursing, Hsin-Sheng College of Medical Care and Management, Taoyuan, Taiwan; 5 Department of Medical Research, Kaohsiung Medical University Hospital, Kaohsiung Medical University, Kaohsiung, Taiwan

## Abstract

Diabetes mellitus is associated with an increased risk of Alzheimer’s dementia and cognitive decline. The cause of neurodegeneration in chronic diabetic patients remains unclear. Changes in brain microglial activity due to glycemic fluctuations may be an etiological factor. Here, we examined the impact of acute ambient glucose fluctuations on BV-2 microglial activity. Biochemical parameters were assayed and showed that the shift from normal glucose (NG; 5.5 mM) to high glucose (HG; 25 mM) promoted cell growth and induced oxidative/inflammatory stress and microglial activation, as evidenced by increased MTT reduction, elevated pro-inflammatory factor secretion (i.e., TNF-α and oxygen free radicals), and upregulated expression of stress/inflammatory proteins (i.e., HSP70, HO-1, iNOS, and COX-2). Also, LPS-induced inflammation was enlarged by an NG-to-HG shift. In contrast, the HG-to-NG shift trapped microglia in a state of metabolic stress, which led to apoptosis and autophagy, as evidenced by decreased Bcl-2 and increased cleaved caspase-3, TUNEL staining, and LC3B-II expression. These stress episodes were primarily mediated through MAPKs, PI3K/Akt, and NF-κB cascades. Our study demonstrates that acute glucose fluctuation forms the stress that alters microglial activity (e.g., inflammatory activation or self-degradation), representing a novel pathogenic mechanism for the continued deterioration of neurological function in diabetic patients.

## Introduction

Diabetes mellitus (DM) is closely associated with pathological alterations in the cerebral microvasculature, which lead to cognitive deficits and an increased risk of Alzheimer’s disease (AD)^1–3^. The brain uses glucose as a primary energy source; thus, glucose metabolism dysfunction may be responsible for cerebral complications in diabetic patients. The symptoms of diabetes, including hyperglycemia, obesity, increased blood triacylglycerol concentration, and insulin resistance, are risk factors that increase the probabilities of synaptic loss, impaired neurogenesis, neuronal death, and eventual cognitive decline^4,5^. Studies have identified several pathophysiological mechanisms in diabetic neurodegeneration, including oxidative stress, mitochondrial dysfunction, and neuroinflammation^2,4^. The cause of cognitive dysfunction and neurodegeneration in diabetic patients remains poorly understood, hence the etiological factors leading to the continued neurological deterioration in DM require additional study.

The progressive neurodegeneration observed in the diabetic brain is likely caused by the long-term effects of diabetes-induced metabolic alterations and dysglycemia, such as hyperglycemia, hypoglycemia, and acute glycemic fluctuations^3,6^. Actually, diabetic neuropathy is closely associated with glucose-induced neurotoxicity resulting from excessive advanced glycation end products (AGEs), osmotic stress eliciting damage to the blood brain barrier (BBB), and the leak of toxic substances leading to neuronal injury and inflammation-related glial activation^3,7,8^. Hyperglycemia is a recognized risk factor for cognitive impairment. Specifically, the amplification of oxidative stress and inflammation by hyperglycemia causes deleterious effects on cerebral function by increasing the production of free radicals and circulating cytokines while impairing antioxidant and innate immune defences^9^. Glycemic variability has been proposed to promote cognitive dysfunction^6,10^; however, the impact of acute glycemic fluctuations between peaks and nadirs on neural cells is less documented. Both upward (postprandial) and downward (interprandial) acute changes in glycemia may enhance neural damage during chronic brain inflammation, and thus enlarge and accelerate the deterioration of cognitive performance in diabetic patients.

Microglia play an important role in diabetic neuropathy. In experimentally-induced diabetic mouse models, microglial proliferation and activation were observed in the brain; in addition, activated microglia largely contributed to neuroinflammatory processes and oxidative stress^11–13^. Thus, the microglial activity (e.g., chronic activation or self-degradation) associated with enhancing neurodestructive effects or withdrawing neurotrophic effects should be a concern in diabetic brains. Microglia are the most susceptible to pathological brain changes, and BBB injury is apparent in diabetes^14^; hence, glycemic variability may easily disturb microglial activity during BBB dysfunction. To the best of our knowledge, the response of microglia to acute glucose fluctuations remains unclear.

In this study, we examined whether cerebral glycemic variability played a crucial role leading to the disturbance of microglial activity using an *in vitro* culture model of murine BV-2 microglial cells. To mimic *in vivo* acute fluctuations in glycemia, we rapidly shifted from normal to high glucose (NG-to-HG) and from high to normal glucose (HG-to-NG). Biochemical parameters and cell fates after glucose shifts were evaluated as a measure of microglial activity. Here we provide reliable data illustrating that the stress ascribed to acute fluctuations in surrounding glucose induces inflammatory activation or self-degradation in microglia.

## Results

### An NG-to-HG shift increases microglial proliferation and GLUT2 expression

**Figure 1:**
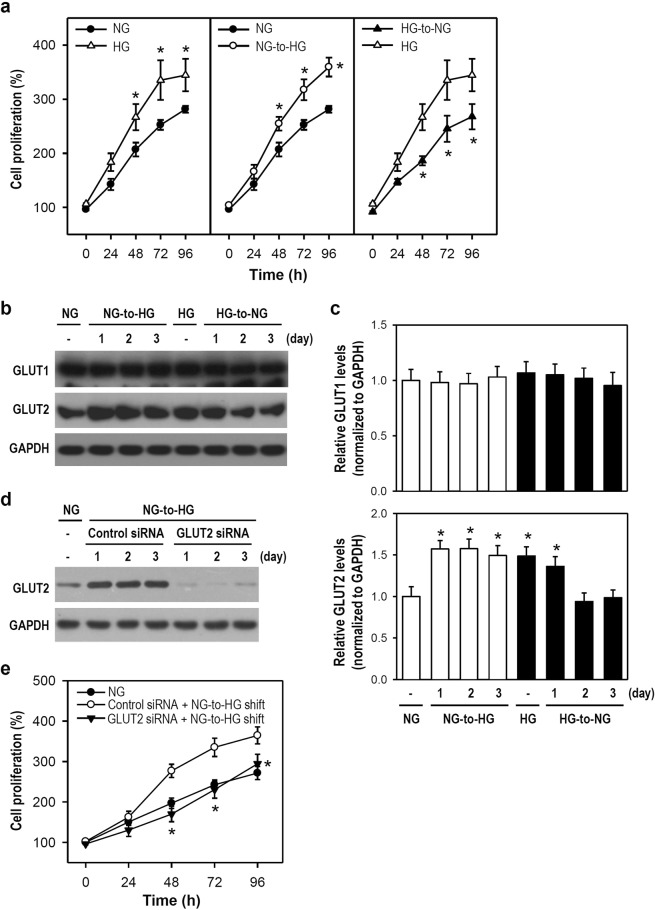
An NG-to-HG shift increases cell proliferation and the protein expression of GLUT2 but not GLUT1 in cultured BV-2 microglia. (**a**) Two BV-2 cell lines were cultured separately in NG and HG media (i.e., constant NG or HG). Some cells were then exposed to a glucose shift (i.e., NG-to-HG or HG-to-NG). At the incubation time points indicated, cells were harvested and subjected to the MTT reduction assay. Cell proliferation was calculated as a percentage relative to the cell number at 0 h (100%). Each point represents the mean ± SEM from five independent experiments performed in triplicate. *p < 0.05 versus constant NG group (left and middle panels) or constant HG group (right panel) at the same time point. (**b**,**c**) The media used to maintain NG- and HG-cultured cells were replaced by HG and NG media, respectively, and cells were further incubated for 1, 2, or 3 days. After harvest, cell lysates were subjected to western blotting for GLUT1 and GLUT2 expression (for uncropped images of the blots please see Supplementary Information). GAPDH served as a protein loading control. The histograms illustrate relative levels obtained by quantifying the band intensity. The corresponding GAPDH levels were used for normalization, and the NG-cultured cell group was assigned a value of 1. Data are represented as mean ± SEM from four independent experiments. *p < 0.05 versus constant NG group. (**d**,**e**) Transient siRNA transfection was performed to silence GLUT2 expression and then the efficacy was determined by western blotting (for uncropped images of the blots please see Supplementary Information). A non-related, scrambled siRNA was used as a control. One day after transfection, the NG medium was replaced by fresh medium with the NG-to-HG shift simultaneously. Cells were cultured continuously for several days. At the indicated time points, cells were harvested for determining the intracellular GLUT2 level or cell proliferation. Each point on the line chart represents the mean ± SEM from four independent experiments performed in triplicate. *p < 0.05 versus the group of control siRNA plus NG-to-HG.

Alterations in the brain environment can trigger neural cell reactivity, followed by adaptation or maladaptation. Once the BBB is damaged, brain glycemic variability can disturb microglial reactivity. We first examined whether glucose fluctuations affect the growth profile of microglia. Two BV-2 cell lines were separately cultured in NG and HG media. As expected, cells incubated in constant HG conditions exhibited higher proliferation than cells cultured in constant NG conditions. NG-cultured cells exposed to an NG-to-HG shift showed a substantial increase in proliferation when compared with cells under constant NG conditions; however, HG-cultured cells receiving an HG-to-NG shift showed a marked decrease in proliferation when compared with cells under constant HG conditions (Fig. 1a and Supplementary Fig. 1). Subsequently, we investigated whether an adaptive change in the expression of GLUT proteins occurs when microglia experience glucose fluctuations. The expression of GLUT2, but not GLUT1, was increased and decreased in response to NG-to-HG and HG-to-NG shifts, respectively (Fig. 1b,c). Regrettably, GLUT5, which is known to be specific for microglia, did not respond to glucose fluctuations (Supplementary Fig. 2). When GLUT2 was knockdowned by RNAi (i.e., GLUT2 siRNA) in NG-cultured cells, no increased GLUT2 expression was found after an NG-to-HG shift (Fig. 1d). Also, a similar proliferation pattern was observed between constant NG-cultured cells and GLUT2-silenced NG-cultured cells receiving an NG-to-HG shift (Fig. 1e). Therefore, our findings suggest that an NG-to-HG shift causes an adaptive increase in microglial proliferation that positively correlates with GLUT2 upregulation. The upregulation in GLUT2 protein may be essential for the machinery that increases microglial proliferation after experiencing an NG-to-HG shift.

### An NG-to-HG shift activates microglia, leading to an upregulation of stress and inflammatory proteins

**Figure 2:**
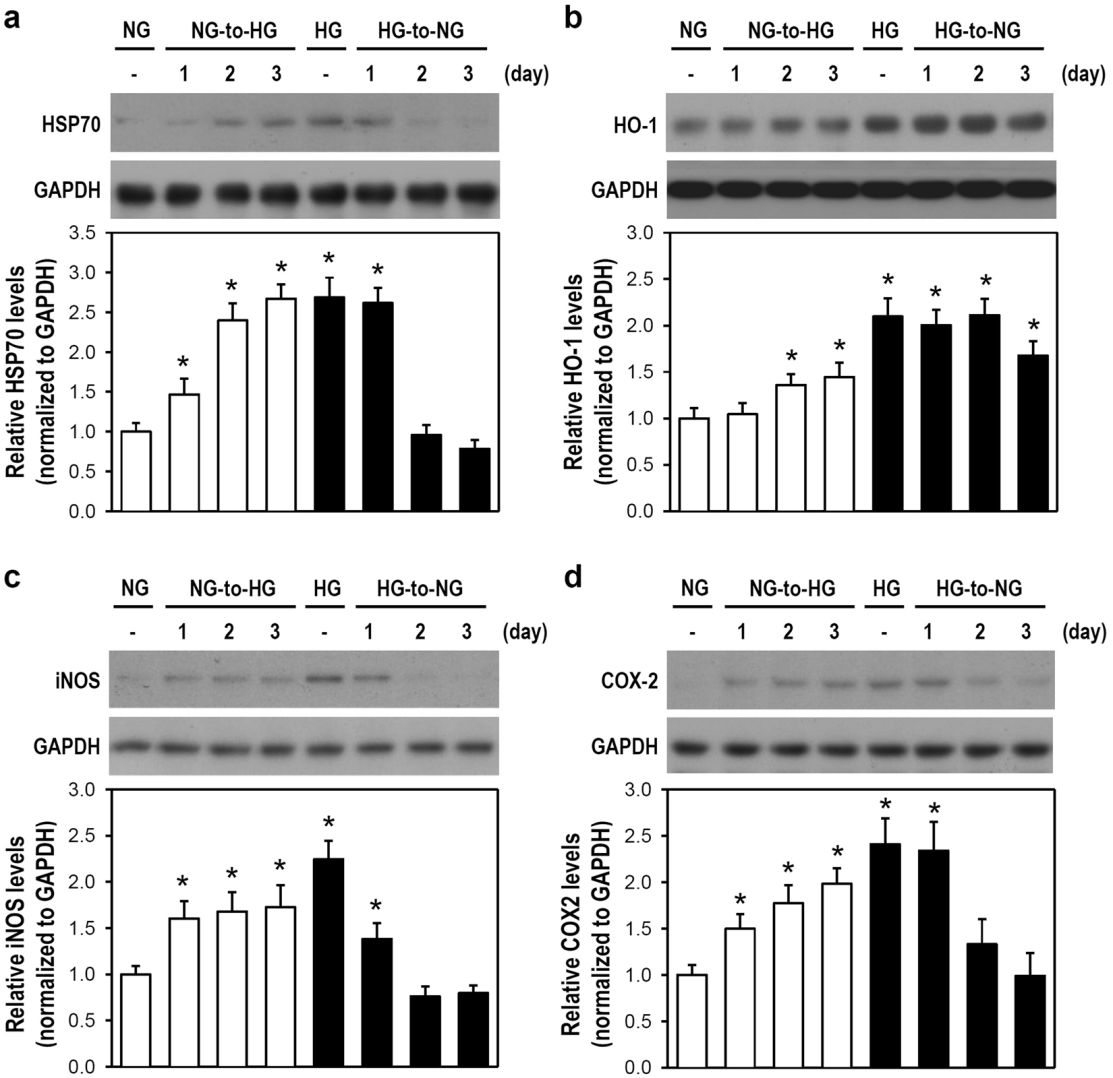
An NG-to-HG shift leads to the induction of HSP70, HO-1, iNOS, and COX-2 proteins in cultured BV-2 microglia. (**a**–**d**) The media of BV-2 cells assigned as NG- and HG-cultured cells were replaced by HG and NG media, as indicated. Next, cultures were consecutively incubated for 1, 2, or 3 days. After cell harvest, western blotting was performed to detect HSP70 (**a**), HO-1 (**b**), iNOS (**c**) and COX-2 (**d**) expression (for uncropped images of the blots please see Supplementary Information). GAPDH served as a protein loading control. Quantitative analysis of the data was performed for each protein. The histograms illustrate the relative expression levels obtained by quantification of western blot band intensity using densitometry and by normalization to the corresponding GAPDH level. The NG-cultured cell group was assigned a value of 1. Data are represented as mean ± SEM from four or five independent experiments. *p < 0.05 versus constant NG group.

Although glucose fluctuations trigger cellular/molecular changes that enable microglia to adapt to the environment, we speculated that these fluctuations act as a stressor that elicits dramatic changes in microglial activity. Stress responses from a cell adapting to unfavorable environmental conditions are typically mediated via stress proteins. We examined HSP70 and HO-1 because both are cytoprotective and stress-inducible proteins in several experimental models, including global ischemia and oxidative stress-induced gliotoxicity^15,16^. Cells exposed to an NG-to-HG shift showed upregulated levels of HSP70 and HO-1 within 3 days when compared with cells in constant NG conditions. Also, a reverse in the elevation of stress proteins was observed after an HG-to-NG shift in HG-cultured cells (Fig. 2a,b).

The appearance of stress proteins implies that stress is occurring. Thus, microglial activity in response to acute glucose fluctuations was evaluated. Activation is essential for a cell’s response to external stimuli and is typically featured by protein neo-synthesis or elevated enzyme activity to accelerate and amplify intracellular biochemical reactions. Given that iNOS and COX-2 upregulation correlates with pathological stress, such as neuroinflammation characterized by microglial activation and the production of inflammatory factors^17^. The neo-synthesis of these inflammatory proteins was assayed as a marker of microglial activation. NG-cultured cells exposed to an NG-to-HG shift showed iNOS and COX-2 upregulation when compared with cells in constant NG conditions. Furthermore, a considerably gradual decline in the upregulation of iNOS and COX-2 proteins was observed after HG-cultured cells were exposed to an HG-to-NG shift (Fig. 2c,d). These findings suggest that an acute fluctuation from NG to HG forms a stress that induces microglial activation, and this activation is reversed by an HG-to-NG shift.

### An NG-to-HG shift induces the release of pro-inflammatory factors from activated microglia

**Figure 3:**
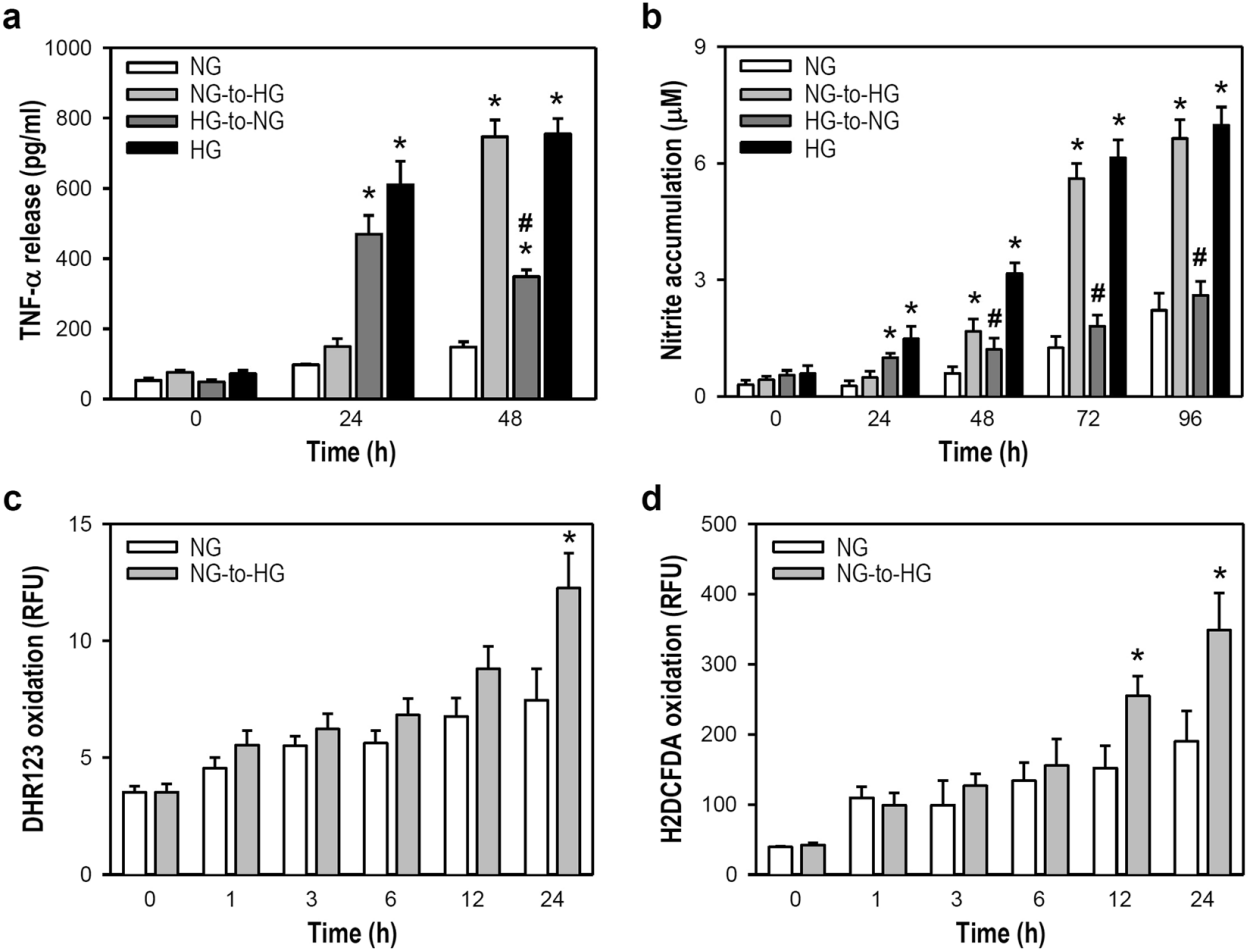
An NG-to-HG shift increases the production of TNF-α, NO, peroxides, and ROS in cultured BV-2 microglia. (**a**,**b**) At an assigned time point of 0 h, the culture media used to maintain NG- and HG-cultured cells was replaced by fresh media with or without a shift in glucose concentration, as indicated. To avoid any influence of different cell proliferation rates on the assays, ARA-C (2 µg/ml) was added to all treatment groups. Cell media was collected at the indicated time points for the measurement of TNF-α using ELISA (**a**) or NO using an assay of the Griess reaction to determine nitrite accumulation (**b**). Data are represented as mean ± SEM from five independent experiments performed in triplicate. *p < 0.05 versus constant NG group at the same time point; ^#^*p* < 0.05 versus constant HG group at the same time points. (**c**,**d**) Culture media was replaced by fresh media at the assigned time point of 0 h with or without an NG-to-HG shift. ARA-C was added to all treatment groups to eliminate the influence of different cell proliferation rates on the detected parameters. At different time points within 0−24 h, cell-permeable fluorogenic probes, DHR-123 (**c**) and H2DCFDA (**d**), were employed individually. DHR-123 monitors the production of ROS, such as peroxide and peroxynitrite. H2DCFDA serves as a tool to detect intracellular ROS, reflecting the degree of overall oxidative stress. Data are represented as mean ± SEM from four independent experiments performed in triplicate. *p < 0.05 versus constant NG group at the same time point. RFU: relative fluorescence unit.

Chronic hyperglycemia and glycemic variability can induce pro-inflammatory conditions^18^; thus, inflammation plays a crucial role in diabetes-related neurodegeneration. We examined whether the microglial activation elicited by an NG-to-HG shift elevates the production of pro-inflammatory factors. To minimize the influence of different cell proliferation rates on assay parameters, cytosine arabinoside (ARA-C, 2 μM), which has no cell toxicity or oxygen free radical induction, was used (Supplementary Fig. 3). NG-cultured cells exposed to an NG-to-HG shift showed an obvious increase in TNF-α production at 48 h when compared with cells under constant NG conditions. The HG-to-NG shift in HG-cultured cells did not completely reduce TNF-α levels to those of NG-cultured cells in constant NG conditions within 48 h; however, a markedly lower TNF-α level was detected at 48 h in HG-cultured cells exposed to an HG-to-NG shift when compared with cells under constant HG conditions (Fig. 3a). The NG-to-HG shift caused an evident increase in NO production after 48 h when compared to constant NG conditions, while an HG-to-NG shift in HG-cultured cells effectively slowed NO production nearly to that of NG-cultured cells under constant NG conditions (Fig. 3b). The NG-to-HG shift also increased the production of oxygen free radicals when compared with constant NG conditions. A markedly higher formation of peroxides was observed at 24 h (Fig. 3c); likewise, the ROS production was clearly increased at 12 and 24 h (Fig. 3d). These results indicate a pro-inflammatory role of acute NG-to-HG fluctuation in the immune activation of microglia, as evidenced by increased production of TNF-α, NO, and oxygen free radicals.

### Glucose fluctuations activate MAPKs and PI3K/Akt signaling pathways and alter NF-κB activity in microglia

**Figure 4:**
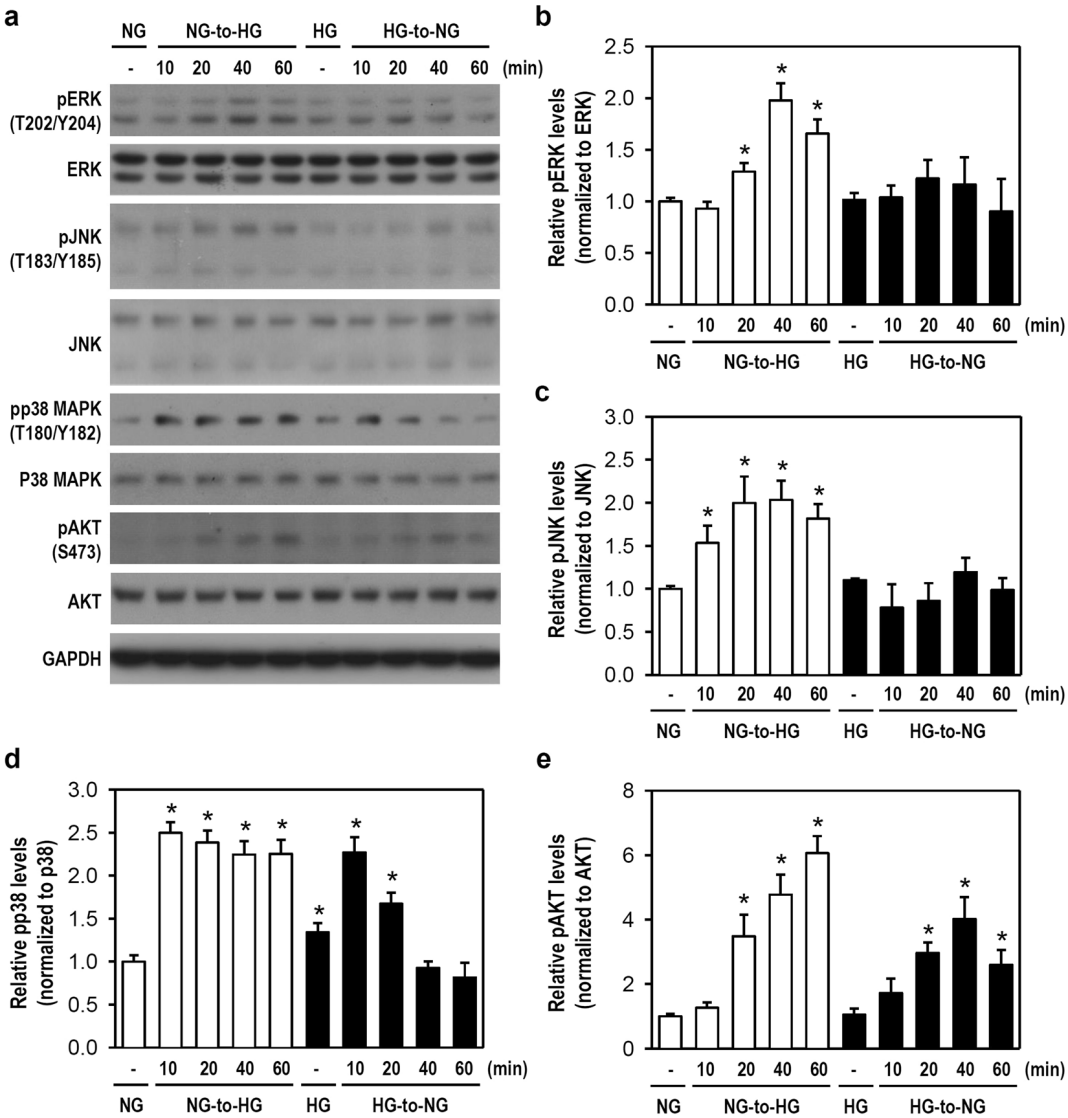
A Shift of NG-to-HG or HG-to-NG activates the signaling kinases of MAPKs and Akt in cultured BV-2 microglia. (**a**) The media of BV-2 cells assigned as NG- and HG-cultured cells was simply renewed using fresh NG and HG media or replaced by HG and NG media, respectively. Next, cultures were incubated for 10, 20, 40, or 60 min. After harvest, western blotting was performed using specific antibodies for pERK, ERK, pJNK, JNK, pp38, p38, pAkt, and Akt (for uncropped images of the blots please see Supplementary Information). GAPDH served as a protein loading control. The representative blots from one of four independent experiments are presented. (**b**–**e**) The expression levels of pERK, pJNK, pp38, and pAkt were quantified. The histograms show the relative levels obtained using densitometry to quantify western blot band intensity, followed by normalization to the corresponding total protein level. The quantitative values were expressed relative to the constant NG group (assigned a value of 1). Data are represented as mean ± SEM from four independent experiments. *p < 0.05 versus constant NG group.

**Figure 5:**
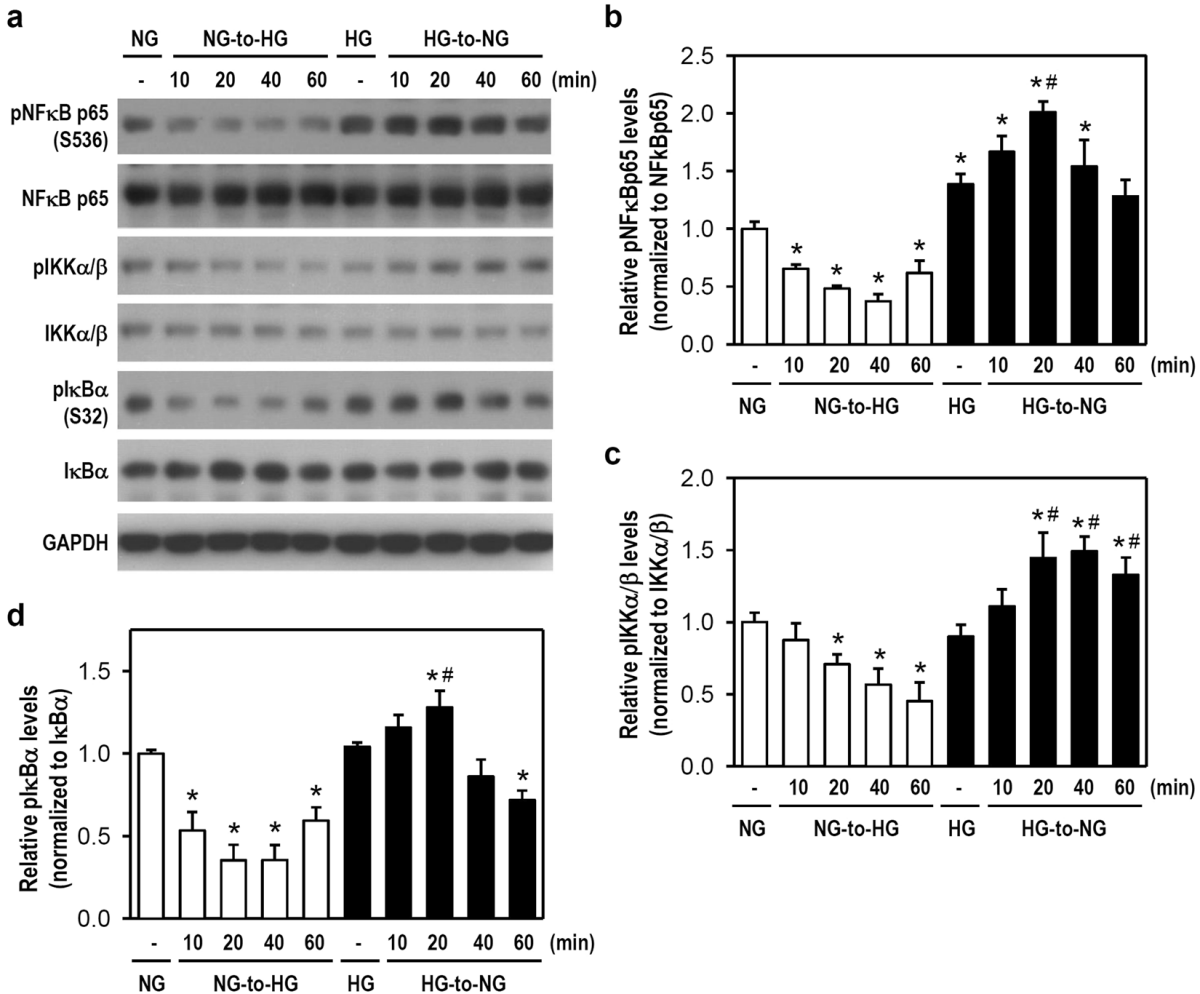
Shifts of NG-to-HG and HG-to-NG differentially alter NF-κB activity in cultured BV-2 microglia. (**a**) The media of NG- and HG-cultured cells was simply renewed using fresh media or replaced by HG and NG media, as indicated. Next, cultures were incubated for 10, 20, 40, or 60 min. After harvest, western blotting was performed for pNF-κB p65, NF-κB p65, pIKKα/β, IKKα/β, pIκBα, and IκBα (for uncropped images of the blots please see Supplementary Information). GAPDH served as a protein loading control. The representative blots extracted from one of four independent experiments are presented. (**b**–**d**) The expression levels of pNF-κB p65, pIKKα/β, and pIκBα were quantified. The histograms show the relative levels obtained using densitometry to quantify western blot band intensity, followed by normalization to the corresponding total protein level. The quantitative values were expressed relative to the level of the constant NG group (assigned a value of 1). Data are represented as mean ± SEM from four independent experiments. *p < 0.05 versus constant NG group; ^#^*p* < 0.05 versus constant HG group.

Next, we examined the possible signaling cascades involved in our aforementioned results. Previous studies suggested that MAPKs participate in stress-associated cell activation and inflammation, whereas PI3K/Akt is involved in glucose metabolism and cell proliferation^19–21^. NF-κB plays a role in the pathogenesis of inflammatory diseases and is a crucial transcription factor for glial cell function^22,23^. So, the phosphorylation state of ERK, JNK, p38, and Akt as well as NF-κB activity were assessed in microglia after acute glucose fluctuations. An NG-to-HG shift in NG-cultured cells markedly increased the phosphorylation levels of ERK, JNK, p38, and Akt within 1 h when compared with constant NG conditions (Fig. 4a–e). Notably, the HG-to-NG shift in HG-cultured cells only activated p38 and Akt signalings (Fig. 4a,d,e). Additionally, levels of phosphorylated NF-κB p65 rapidly declined as early as 10 min following an NG-to-HG shift in NG-cultured cells, whereas an HG-to-NG shift in HG-cultured cells markedly elevated NF-κB p65 phosphorylation at 20 min (Fig. 5a,b). These NF-κB p65 phosphorylation events temporally parallel the changes in phosphorylated IKKα/β and IκBα levels (Fig. 5a,c,d). Our findings suggest that acute glucose fluctuations rapidly activate intracellular signaling cascades, particularly p38 and Akt, that trigger resting microglia to an active phenotype. NF-κB immediately reacts to glucose shifts and thus is a crucial factor of microglial activation even though its activity negatively correlates with the glucose concentration transition.

### An NG-to-HG shift induces oxidative/inflammatory stress in microglia via MAPKs, PI3K/Akt, and NF-κB pathways

**Figure 6:**
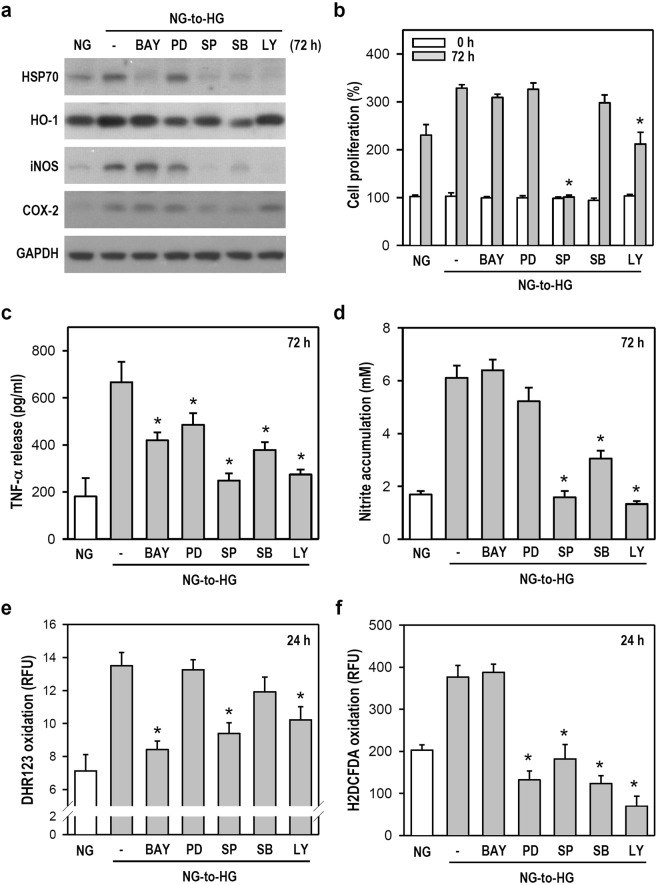
Various signaling pathway inhibitors differentially suppress the NG-to-HG shift-induced upregulation of protein expression (i.e., iNOS, COX-2, HSP70, and HO-1), cell proliferation, and pro-inflammatory factors in cultured BV-2 microglia. (**a**) NG-cultured cells were treated with vehicle or various inhibitors (i.e., 5 µM BAY11-7082, 20 µM PD98059, 20 µM SP600125, 20 µM SB203580, and 20 µM LY294002) for 30 min prior to the culture media being replaced from NG to HG. Cultures were continuously incubated in HG medium containing these inhibitors for another 72 h. After harvest, western blotting was performed for HSP70, HO-1, iNOS, and COX-2 levels (for uncropped images of the blots please see Supplementary Information). GAPDH served as a protein loading control. The quantitative analyses for HSP70, HO-1, iNOS, and COX-2 are shown in Supplementary Fig. 4. (**b**–**f**) Similar to the treatments in panel a, cultures at 24 or 72 h were subjected to biochemical assays for cell proliferation (**b**), TNF-α release (**c**), nitrite accumulation (**d**), and productions of peroxides (**e**) and ROS (**f**). To minimize the influence of different cell proliferation rates between NG and NG-to-HG groups on TNF-α, nitrite, peroxides, and ROS measurements, ARA-C was added. To quantitate cell proliferation in panel b, the cell number for each group at 0 h was assigned a value of 100%. Data are represented as mean ± SEM from four or five independent experiments performed in triplicate. *p < 0.05 versus NG-to-HG group treated with vehicle.

To determine the underlying signaling cascades that mediate stress-related activation responses, signaling pathway inhibitors, including BAY11-7082 (BAY; IκB phosphorylation inhibitor), PD98059 (PD; MEK/ERK inhibitor), SP600125 (SP; JNK inhibitor), SB203580 (SB; p38 inhibitor), and LY294002 (LY; PI3K inhibitor), were used in microglia. Compared to vehicle-treated cells exposed to an NG-to-HG shift, we found the following effects: 1) BAY, SP, SB, and LY effectively reduced HSP70 expression; 2) PD, SP, and SB substantially decreased HO-1 expression; 3) SP, SB, and LY markedly diminished iNOS expression; and 4) SP and SB notably weakened COX-2 expression (Fig. 6a and Supplementary Fig. 4). These results indicate that the NG-to-HG shift-induced expression of both stress and inflammatory proteins is mediated by a cooperation of distinct signaling cascades. HSP70 induction is mediated by NF-κB, whereas the PI3K/Akt pathway closely correlates with the neo-synthesis of HSP70 and iNOS. MAPKs, especially JNK, are major cascades mediating the upregulation of all inducible proteins. Regarding NG-to-HG shift-induced microglial proliferation, the JNK and PI3K/Akt are the main kinases involved, as evidenced by the SP- and LY-mediated inhibition of proliferation (Fig. 6b). All inhibitors clearly reduced NG-to-HG shift-induced TNF-α production (Fig. 6c). Consistent with the results above (Fig. 6a; iNOS blot), NO production closely correlated with the activation of JNK, p38, and PI3K/Akt pathways after an NG-to-HG shift (Fig. 6d). Moreover, the NG-to-HG shift-induced formation of peroxides was associated with the NF-κB, JNK, and PI3K/Akt pathways, as demonstrated by the effectiveness of BAY, SP, and LY (Fig. 6e). All used inhibitors, except BAY, clearly repressed the elevation of intracellular ROS induced by an NG-to-HG shift, thus indicating that NF-κB is not involved (Fig. 6f). These data suggest that among the examined signaling pathways, JNK is the most important kinase in the early response to an NG-to-HG shift because its activation is detectable in all episodes of oxidative/inflammatory stress.

### LPS-induced microglial inflammation is strengthened by an NG-to-HG shift

**Figure 7:**
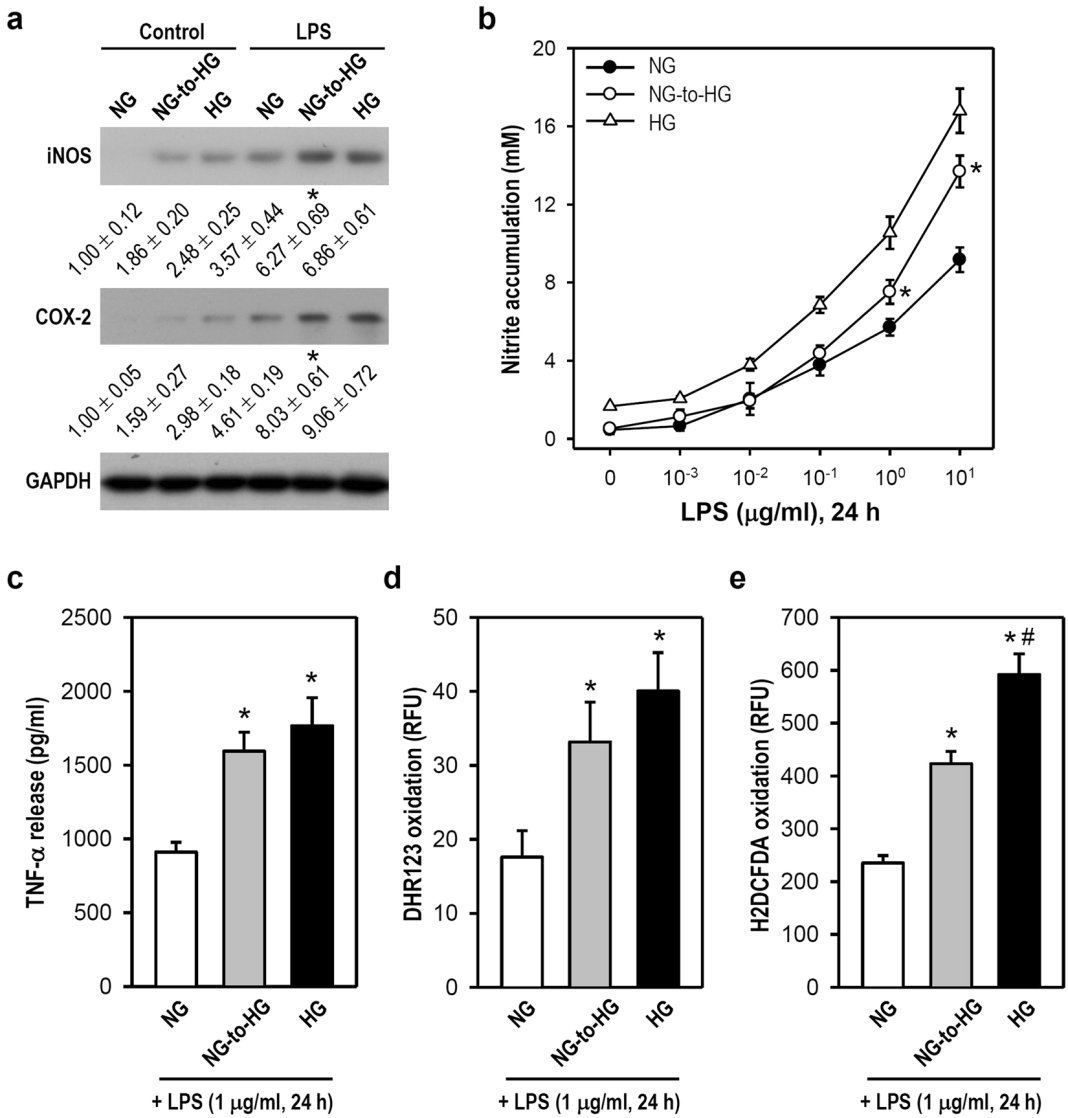
The NG-to-HG shift enhances LPS-induced inflammation in cultured BV-2 microglia. (**a**) As indicated, both NG- and HG-cultured cells were simply treated with or without LPS (1 µg/ml); also, an NG-to-HG shift with or without LPS treatment in NG-cultured cells was performed. After incubation for 24 h, western blotting was used to detect iNOS and COX-2 levels (for uncropped images of the blots please see Supplementary Information). GAPDH served as a protein loading control. After normalization to corresponding GAPDH, iNOS and COX-2 levels were quantified using densitometry and expressed relative to the constant NG group (assigned a value of 1). The mean ± SEM from four independent experiments is shown below the representative blots. *p < 0.05 versus constant NG group treated with LPS. (**b**–**e**) Both NG- and HG-cultured cells as well as the NG-cultured cells receiving an NG-to-HG shift were incubated with different concentrations of LPS (0.001–10 µg/ml; **b**) or single LPS concentration (1 µg/ml; **c**–**e**) for 24 h. ARA-C was added to cultures to avoid the influence of cell proliferation rates. After harvest, cells were subjected to various assays for determining nitrite accumulation (**b**), TNF-α release (**c**), and productions of peroxides (**d**) and ROS (**e**). Data are represented as mean ± SEM from four to five independent experiments performed in triplicate. *p < 0.05 versus constant NG group at equal LPS concentration (**b**) or constant NG group treated with LPS (**c–e**); ^#^*p* < 0.05 versus NG-to-HG group treated with LPS.

We next assessed whether an NG-to-HG shift enhances LPS-induced inflammation in microglia. NG-cultured cells were treated with an NG-to-HG shift or simple medium renewal in the absence or presence of LPS for 24 h. Western blotting analyses showed that the expression of LPS-induced iNOS and COX-2 was substantially enhanced after an NG-to-HG shift when compared with LPS exposure in constant NG conditions (Fig. 7a). In comparison with LPS exposure under constant NG conditions, an NG-to-HG shift evidently increased LPS-induced NO production (≥ 1 μg/ml; Fig. 7b). Also, the NG-to-HG shift promoted LPS-induced inflammatory responses, including TNF-α, peroxides, and ROS productions (Fig. 7c–e). Therefore, these results clearly suggest that an acute fluctuation from NG to HG enlarges LPS-induced inflammation in microglia; in other words, microglia experiencing an NG-to-HG shift easily reach a fully-activated state and produce a greater response in the presence of inflammatory stimuli.

### The HG-to-NG shift induces metabolic stress, causing microglial apoptosis and/or autophagy

**Figure 8:**
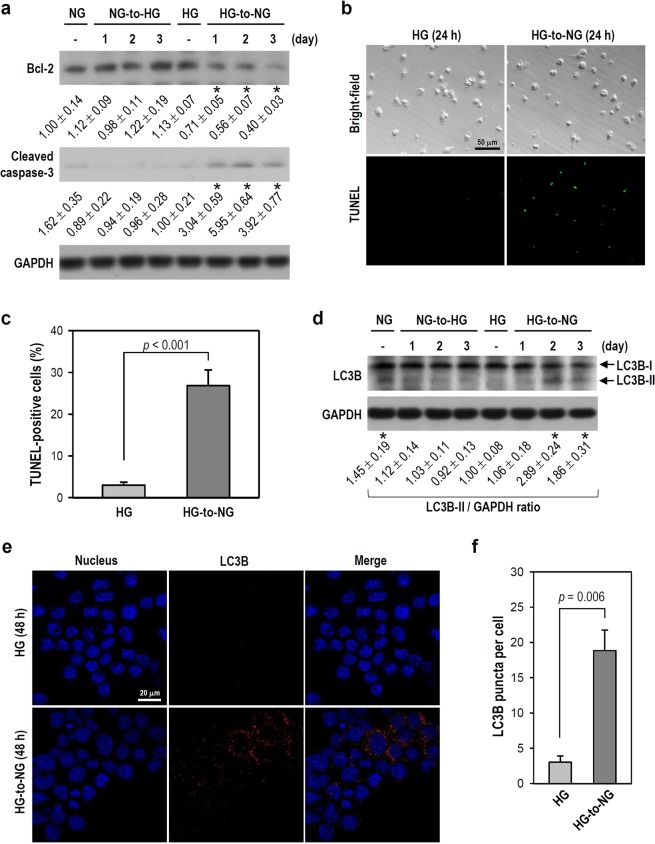
An HG-to-NG shift induces both apoptosis and autophagy in cultured BV-2 microglia. (**a**) The media of BV-2 cells assigned as NG- and HG-cultured cells was simply renewed or replaced by HG and NG media, as indicated. Next, cultures were consecutively incubated for 1, 2, or 3 days. After harvest, western blotting was performed to detect Bcl-2 and cleaved caspase-3 expression (for uncropped images of the blots please see Supplementary Information). The levels of Bcl-2 and cleaved caspase-3 were quantified using densitometry, normalized to corresponding GAPDH, and expressed relative to the level of constant NG or HG group (assigned a value of 1). The mean ± SEM from four independent experiments is shown below the representative blots. *p < 0.05 versus constant HG group. (**b**) A TUNEL assay was performed 24 h after the HG-to-NG shift or simple medium renewal. The representative photos of bright-field and matching TUNEL fluorescence are shown. (**c**) The histogram shows the percentage of TUNEL-positive cells determined by calculating the number of TUNEL-positive nuclei with fluorescence as a proportion of total cell number in the matching bright-field photo. At least 25 sets of bright-field and matching fluorescent photos were examined in each treatment group per experiment. Data are represented as mean ± SEM from three independent experiments. (**d**) Similar to the treatments in panel a, western blotting was used to detect LC3B levels at different time periods (for uncropped images of the blots please see Supplementary Information). After normalization to corresponding GAPDH, quantitative LC3B-II values were represented as mean ± SEM from four independent experiments and are shown below the representative blot. *p < 0.05 versus constant HG group. (**e**) Representative fluorescence photos of LC3B are shown. Cells grown on coverslips were harvested 48 h after simple medium renewal or HG-to-NG shift. Immunostaining was performed using DAPI to recognize the nucleus (blue) and an antibody specific for LC3B (red). (**f**) The histogram shows the quantification of panel e. The numbers of LC3B puncta per cell were counted, and at least 200 cells pooled from three independent experiments were examined in each treatment group. Data are represented as mean ± SEM.

The acute fluctuation from HG to NG is another concern for diabetes-related brain degeneration; hence, we examined microglial activity after an HG-to-NG shift. The deprivation of survival essentials, such as nutrients, can place cells in an adverse situation that triggers apoptosis or autophagy^24^. We found that an HG-to-NG shift in HG-cultured cells decreased Bcl-2 expression (an anti-apoptotic protein) and increased the cleaved form of caspase-3 (a key apoptotic caspase) after 24 h (Fig. 8a). The TUNEL assay, a common method to detect DNA strand breaks resulting from apoptosis, was used. A marked increase in the percentage of TUNEL-positive cells was observed after an HG-to-NG shift when compared with constant HG conditions (Fig. 8b,c). LC3-I is converted to LC3-II through lipidation during autophagy. We observed that the LC3B-II level was evidently increased on days 2 and 3 after HG-cultured cells were exposed to an HG-to-NG shift when compared with cells in constant HG conditions; also, cells maintained in constant NG conditions showed higher LC3B-II expression than cells exposed to an NG-to-HG shift (Fig. 8d). The extent of LC3B-II association with autophagic vesicles was immunocytochemically evaluated and visualized as red fluorescent puncta under a microscope. Quantitatively, more LC3B puncta were observed in HG-cultured cells exposed to an HG-to-NG shift when compared with cells maintained in constant HG conditions (Fig. 8e,f). Together, these data suggest that an acute fluctuation from HG to NG creates metabolic stress. If microglial maladaptation occurs under such a circumstance, apoptosis and/or autophagy ensues soon.

## Discussion

DM leads to cognitive dysfunction and is a major contributor to dementia risk. Although studies have investigated the roles of many factors associated with diabetes-related brain complications, the etiological conditions leading to the pathogenesis of Alzheimer’s dementia have not been fully identified. As demonstrated by many clinical trials, these complications are ascribed to dysglycemia, which has two components: chronic hyperglycemia and acute glycemic fluctuations. The effects of these components on the brain are not well understood; nevertheless, their contribution to neurodegeneration may be due to altered microglial performance, which halts trophic support or exerts toxic effects. The varied microglial activities found in diabetic neuropathy presumably mediate and amplify brain complications^12,13^. Here, we characterized the impact of acute ambient glucose fluctuations on microglial activity and offered new insights into pathological mechanisms and therapeutic considerations for diabetic brain degeneration.

The causes of neurological dysfunction in diabetes can be broadly attributed to BBB leakage combined with cerebral metabolic alterations. The BBB protects brain parenchyma from variations in glucose, which cannot normally cross the BBB without the assistance of carriers or transporters; accordingly, neural cells responsive to glycaemic fluctuations suggest that the BBB integrity is compromised. Actually, compromised BBB integrity has been reported in some diabetic patients and experimental models. For example, BBB destruction occurred in hyperglycemic chorea patients^25^. Compared to non-diabetic patients, white matter lesions were frequently observed in diabetic patients^26^, implying diabetes-induced BBB disruption in clinical practice. In streptozotocin-induced diabetic rats, BBB permeability was enhanced by a decrease in tight junction proteins (i.e., occludin and ZO-1) and an increase in matrix metalloproteinase activity^27^. Additionally, complications commonly associated with diabetes, such as cerebral ischemia, hypertension, and hyperosmolality, disrupt the BBB integrity and thus allow biomolecules that are normally confined to the blood to enter the brain parenchyma^28^. BBB dysfunction is more pronounced in AD patients with peripheral vascular abnormalities, such as diabetes and cardiovascular diseases^29^. Following tight junction disruption, altered transport of biomolecules across the BBB, aberrant angiogenesis, vessel regression, brain hypoperfusion, and inflammatory responses that weaken synaptic plasticity and neuronal survival have been reported in AD patients^7,30^. Thus, cerebral metabolic variations, including glycemic fluctuations, may easily disturb neural cell activity under injured BBB conditions, leading to cognitive disability in the diabetic brain.

GLUTs are essential suppliers of glucose to neurons and glia within the brain. In the brain, GLUT2 serves as a central glucosensor to control food intake and is regulated by energy/glucose status^31^. Notably, we found that the expression level of GLUT2, but not GLUT1, was upregulated in response to ambient alterations from NG to HG. This elevated GLUT2 level might contribute to microglial activation and proliferation. Consistent with previous studies in pancreatic islet β-cells and hepatocytes, GLUT2 levels were passively increased by HG levels, in turn leading to augmented glucose influx and metabolism^32,33^. GLUT2 overexpression in GT1-7 neuroblastoma cells resulted in increased cellular ATP levels at 5 mM or higher glucose levels^34^. Although GLUT5, one of the primary GLUT isoforms in the brain, is specifically expressed in microglia, its regulation remains unclear. It was previously reported that microglial GLUT5 expression did not correlate with prolonged hyperglycemia^35^. In fact, GLUT5 has a much higher affinity for fructose than for glucose and is regarded as a fructose transporter. Fructose feeding in rats increased the expression of both GLUT5 mRNA and protein in the brain^36^. It is plausible that increased microglial GLUT5 expression promotes fructose utilization as an energy source if glucose utilization is impaired or glucose is not available. A study using human brain tissue samples (7 AD and 7 control brains) showed decreased GLUT1 and GLUT3 protein levels but a drastic increase in GLUT2 in the AD brain^37^. We determined that microglial GLUT2 positively correlated with glucose fluctuations; however, its role in the progression of diabetic neurodegeneration remains unclear and needs further exploration (please also see Supplementary information for extensive discussion on brain GLUTs).

Glucose is readily utilized by immunocompetent cells, such as microglia, to generate energy. Thus in this study, we speculate that the upregulation of GLUT2 by an NG-to-HG shift greatly increases glucose uptake/utilization, elevates the activity of the mitochondrial respiratory chain, promotes the production of more oxygen free radicals (e.g., ROS and NO), and leads to the formation of oxidative stress. Other sources of oxidative stress include glucose auto-oxidation, the polyol pathway with ensued depletion of antioxidant reserves, and the formation of AGEs^38^. Indeed, we found increased NO, peroxides and ROS production as well as the expression of the stress-inducible proteins HSP70 and HO-1 after microglia were exposed to an NG-to-HG shift. Stress conditions, such as global ischemia, sublytic NO exposure, or short-term HG exposure (30 mM), can induce HSP70 and/or HO-1 expression in injured neural cells and retinal endothelial cells^15,16,39^. We reasonably consider that an NG-to-HG fluctuation activates oxidative stress in microglia, which in turn induces HSP70 and HO-1 expression for resistance against stress insults.

In addition, the NG-to-HG shift also created inflammatory stress that activated microglia in the present study. Excessive ROS caused by dysglycemia can activate redox-sensitive pro-inflammatory transcription factors such as NF-κB, activator protein-1, and early growth response-1. The genes regulated by these transcription factors are inducible, as observed by an increased expression of various pro-inflammatory cytokines and chemokines (e.g., TNF-α, IL-6, and MCP-1) at the mRNA level in the monocytes and plasma^23,40^. Accordingly, diabetic inflammation occurs. An acute increase in circulating inflammatory cytokine levels as a result of hyperglycemia has been reported in diabetic patients^18^. Similarly, our results showed the activation of microglia after exposure to an NG-to-HG shift, which was accompanied by an inducible expression of iNOS and COX-2 and a marked elevation in TNF-α production. Additionally, NG-to-HG shift-stimulated pro-inflammatory conditions in microglia greatly enhanced LPS-induced inflammation. This illustrates that acute glucose elevation is a pro-inflammatory stimulus that enables microglia to reach a pre-activated or semi-activated state, while the enhancement of LPS-induced inflammation by an NG-to-HG shift might be ascribed to the upregulation of microglial GLUT2, followed by elevated glucose uptake and consumption. Likewise, increasing glucose (5.5 to 22 mM) worsened IL-1β-induced vascular inflammation even though no inflammatory effect was observed in the HG only condition^41^ (please also see Supplementary information for extensive discussion regarding the effect of glucose on LPS-induced inflammation).

With respect to constant NG- or HG-incubation conditions applied to BV-2 microglia, it is reasonable that a stable condition triggers no significant alterations in cell activity. Most likely, protein levels remain almost constant, presenting no detectable changes in the short term. As we expected, a consecutive maintenance for 1−3 days following a simple medium renewal (e.g., NG-to-NG or HG-to-HG) did not disturb the baseline expression levels (day 0) of all examined proteins in individual NG or HG circumstances (Supplementary Fig. 5).

Regarding the immediate signaling pathways that contribute to NG-to-HG shift-elicited oxidative/inflammatory stress, we found that MAPKs and PI3K/Akt mediated most stress episodes in microglia. Hyperglycemia-induced mitochondrial ROS can stimulate PKC, JNK, and p38 cascades, which amplify inflammation^42^. Activated p38 is important for cytokine synthesis and release, and JNK inhibition reduces the LPS-induced activity of inflammatory mediators in microglia^19,43^. In diabetes, AGEs activate the MEK/ERK, PI3K/Akt, and NF-κB pathways via RAGE, resulting in the upregulation of pro-inflammatory signals in an *in vitro* AD model using a microglial cell line^8^. As for inducible proteins, including HSP70, HO-1, iNOS, and COX-2, studies using animal and cell culture models under stress conditions have noted that signaling kinases and factors, such as MAPKs, PI3K/Akt, and NF-κB, mediate expression at the mRNA and protein levels^19,44–47^. Similar to past studies examining the effect of HG on cell proliferation^48,49^, we presented that an NG-to-HG shift stimulated microglial growth via JNK and PI3K pathways. Additionally, previous studies showed that the induction of iNOS or COX-2 is associated with active NF-κB^22,23^; however, in this study, we did not detect this association, nor did we find any inhibition of iNOS or COX-2 expression by increasing the dosage of BAY (Supplementary Fig. 6). By comparing the effectiveness of all signaling pathway inhibitors, we concluded that the JNK cascade is the most influential pathway contributing to oxidative/inflammatory stress and microglial activation following acute glucose fluctuations.

Cells respond to metabolic challenges, such as nutrient deprivation, by downregulating energy-consuming processes, such as proliferation and protein synthesis, and stimulating catabolic processes, such as autophagy. In cases of maladaptation, necrosis or apoptosis is a common occurrence. Autophagy upon glucose withdrawal was induced in cardiomyocyte-derived H9c2 cells, human hepatoma cells, and Chinese hamster ovary (CHO) cells^50^. The death of CHO cells triggered by glucose deprivation stimulated autophagic and apoptotic pathways^24^. Consistent with past findings, we observed increased caspase-3 cleavage and LC3B-II expression in microglia after exposure to an HG-to-NG shift. This clearly suggests that partial glucose deprivation creates metabolic stress that promotes both apoptotic and autophagic signal induction. Moreover, Bcl-2, an anti-apoptotic factor, inhibited autophagy *in vivo* and *in vitro*^51^. As expected, we found that reduced Bcl-2 expression was accompanied by the occurrence of apoptosis and autophagy after exposure of microglia to an HG-to-NG shift.

Following partial glucose deprivation, phosphorylated p38, Akt, and NF-κB were observed before the onset of apoptosis and autophagy. Apparently, active p38, Akt, and NF-κB cascades might be largely responsible for these self-degradative processes. As shown in previous studies, p38 is activated by a wide variety of environmental, oxidative, metabolic, and genotoxic stresses, which can induce both apoptosis and autophagy; even, p38 plays a key role in crosstalk between autophagy and apoptosis^52^. Additionally, elevated IKK/NF-κB activity has been reported to positively correlate with the activation of both apoptosis and autophagy. For example, NF-κB activation was induced by apoptotic stimuli, such as DNA damaging agents and ultraviolet radiation. In conditions of hyperglycemia or deprivation of amino acids and serum, autophagy was activated by the IKK/NF-κB pathway coupled to beclin-1 gene expression^50^. With respect to Akt, several lines of evidence suggest that glucose deprivation induces Akt activation^20,53,54^. From our results, Akt activation by an HG-to-NG shift might relate to compensatory feedback in response to an acute decline in ambient glucose; also, elevated Akt activity aims to reduce HG-to-NG shift-induced microglial degradation because PI3K/Akt is well known to promote cell survival and negatively regulate autophagy and apoptosis in different cell types^55^. We conjecture that activated p38 and NF-κB, but not Akt, play roles in mediating the cellular/molecular reactions needed for driving apoptosis and autophagy in microglia. Further studies are needed to elucidate this concept.

The energy depletion resulting from cells incubated in a glucose-free condition leads to an increase in the AMP/ATP ratio, which is sensed by AMP-activated protein kinase (AMPK) to induce autophagy^56^. However, we did not observe AMPK phosphorylation soon after microglial exposure to the HG-to-NG shift (Supplementary Fig. 7). Presumably, partial glucose deprivation (25 to 5.5 mM) in microglia is incapable of inducing a marked alteration of the AMP/ATP ratio that is sufficient to activate AMPK. Indeed, compared to the control (25 mM glucose), human tumor cells incubated under low glucose conditions (1 mM) showed no change in AMPK activity^57^, implying that AMPK is likely activated under glucose levels less than 1 mM or full glucose deprivation.

Glucose fluctuation surely accompanies the change of osmolality. Accordingly, high mannitol was included as a hyperosmolar control to test the impact of osmotic fluctuations per se on microglial activity. In this study, NG-to-HM shift-induced hyperosmolarity was unable to copy the effects we observed in BV-2 cultures that underwent an NG-to-HG shift, whereas the HG-to-HM shift could imitate the effects of the HG-to-NG shift on cell activity (Supplementary Fig. 8). Thus, the specific changes in microglial activity responding to NG-to-HG or HG-to-NG shifts might be ascribed not to osmotic fluctuations but to glucose-related metabolic alterations. In addition, to strengthen the novelty and verity of this study, several critical experiments related to glucose fluctuations were performed once more using primary rat microglial cultures, and the results were still reproducible and significant (Supplementary Fig. 9).

## Conclusions

Overall, we determined that an NG-to-HG shift activates microglia, leading to cell growth and oxidative/inflammatory stress, whereas an HG-to-NG shift causes metabolic stress that activates microglial apoptosis and/or autophagy (Supplementary Fig. 10). The stress episodes are primarily mediated via the MAPK, PI3K/Akt, and NF-κB pathways. Increasing studies suggest that glycemic variability in the cerebral microvasculature closely correlates with the pathogenesis of diabetic cognitive decline and AD. Our data support this correlation and uncover how glycemic fluctuations disturb microglial activity if the BBB is disrupted. Altered microglial activity, such as inflammatory activation or self-degradation, may be an etiological factor for diabetic neurodegeneration. Future strategies to stabilize glycemic levels or microglial activity in the brain may be of great benefit for dementia therapy.

## Methods

### Chemicals, antibodies, and reagents

Tumor necrosis factor-α (TNF-α) ELISA kit was purchased from R&D Systems (Minneapolis, MN, USA). Terminal deoxynucleotidyl transferase-mediated dUTP nick end labelling (TUNEL) kit was purchased from BioVision Inc. (Mountain View, CA, USA). Phospho-ERK (pERK), pJNK, pp38, pAkt, pNF-κB p65, pIKKα/β, pIκBα, ERK, JNK, p38, Akt, NF-κB p65, IKKα/β, IκBα, inducible nitric oxide synthase (iNOS), cyclooxygenase-2 (COX-2), heat shock protein 70 (HSP70), and cleaved caspase-3 antibodies were purchased from Cell Signaling Technology (Beverly, MA, USA). Heme oxygenase-1 (HO-1) and light chain 3B (LC3B) antibodies were purchased from Enzo Life Sciences (Farmingdale, NY, USA). Bcl-2, GAPDH, glucose transporter type 1, 2 and 5 (GLUTs 1, 2 and 5) antibodies, BAY11-7082 agent, and small interfering RNA (siRNA) were purchased from Santa Cruz Biotechnology (Santa Cruz, CA, USA). Alexa Fluor 568-conjugated IgG and HRP-conjugated secondary antibody were obtained separately from Life Technologies (Carlsbad, CA, USA) and Jackson ImmunoResearch Laboratories (West Grove, PA, USA). 2′7′-Dichlorodihydrofluorescein diacetate (H2DCFDA) and dihydrorhodamine-1,2,3 (DHR-123) were purchased from Molecular Probes (Invitrogen, Carlsbad, CA, USA). PD98059, SP600125, SB203580, and LY294002 agents were obtained from Selleckchem (Houston, TX, USA). Culture reagents were obtained from Gibco-BRL/Invitrogen (Carlsbad, CA, USA). All chemicals were from Sigma-Aldrich (Saint Louis, MO, USA) unless stated otherwise.

### Cell cultures and glucose concentration shift

The mouse microglial BV-2 cell line was cultured in Dulbecco’s modified Eagle’s medium (DMEM) supplemented with 10% heat-inactivated fetal bovine serum (FBS), 2 mM L-glutamine, 1 mM sodium pyruvate and 1% penicillin/streptomycin at 37 °C in a humidified atmosphere of 95% air/5% CO_2_. Confluent cultures were passaged twice each week by trypsinization. Before proceeding with this study, BV-2 microglia were separated into two lines: normal glucose (NG; 1 g/L, 5.5 mM)-cultured cells and high glucose (HG; 4.5 g/L, 25 mM)-cultured cells. In the experiments involving acute glucose fluctuations, cell media was replaced by fresh media with a shift of NG-to-HG or HG-to-NG. An NG-to-HG shift indicates that the media for NG-cultured cells was replaced by HG media, whereas the HG-to-NG shift means that the media for HG-cultured cells was changed to NG media. A constant NG or HG condition signifies that the media was renewed without concentration alteration. Cells were also incubated in a high mannitol concentration (HM, 5.5 mM glucose plus 19.5 mM mannitol) to provide a purely hyperosmolar control. In addition, primary rat microglia cells were also prepared. Please see Supplementary information for details.

### RNA interference (RNAi) using siRNA

Prior to transfection, cultures were maintained in antibiotic-free medium for 18−24 h. Cells in 6-well culture plates were transfected with 100 pmol of siRNA duplex using Lipofectamine 2000 and Opti-MEM (Invitrogen, Carlsbad, CA), as per the manufacturer’s protocol. After incubating the transfected cells for 6 h, culture media was replaced by fresh media containing 10% FBS. Western blotting was used for determining the siRNA knockdown effect on target protein. All siRNA oligos were designed and synthesized by Santa Cruz Biotechnology. Mouse GLUT2 siRNA (sc-35496) is a pool of 3 target-specific 19–25 nt siRNAs. The specific sequences are as follows: i) GLUT2 siRNA-1 sense, 5′-CUGCUCACAUAGUCACUAUTT-3′ and antisense, 5′-AUAGUGACUAUGUGAGCAGTT-3′; ii) GLUT2 siRNA-2 sense, 5′-CUACAACUGCUAUCUAGAUTT-3′ and antisense, 5′-AUCUAGAUAGCAGUUGUAGTT-3′; iii) GLUT2 siRNA-3 sense, 5′-CUUCAGUCUUUGUGUUGUATT-3′ and antisense, 5′-UACAACACAAAGACUGAAGTT-3′. Control siRNA (sc-37007) is a non-targeting 20–25 nt siRNA designed as a negative control and leads to no degradation of any known cellular mRNA. The manufacturer does not offer the sequence of scrambled control siRNA.

### Measurements of cell proliferation and viability

Cell proliferation was determined using a tetrazolium-based colorimetric assay (MTT), which measures only *in vitro* living cells and reveals the results related to the number of viable cultured cells. MTT (0.5 mg/mL) dissolved in PBS was added to the cells for 2 h of incubation at 37 °C. After removing MTT-containing medium, the produced purple formazan was dissolved in dimethyl sulfoxide and then measured at 570 nm against a 620-nm reference wavelength (ΔOD, OD: optical density) using a Multiskan microplate photometer (Thermo Fisher Scientific, Waltham, MA, USA). Additionally, a trypan blue exclusion assay was applied to calculate the number of viable cells, which serves as a direct method to measure cell proliferation. Initially, cells were seeded into 24-well plates at a density of 1 × 10^4^ cells per well and exposed to an acute glucose shift the next day. At the indicated time points, cells were removed from the culture plates by trypsin-EDTA and stained with trypan blue for the cell count in Cellometer Auto T4 Cell Counter (Nexcelom Bioscience, Lawrence, MA, USA). For the cell viability assay, cell survival was determined by the VisionBlue™ Quick Cell Viability Fluorometric Assay Kit (BioVision, Mountain View, CA, USA) according to the manufacturer’s instructions. For details, please see Supplementary information.

### Measurement of nitrite accumulation

Since NO itself is unstable, NO production was determined by measuring nitrite, a stable oxidation product of NO. The extent of nitrite released into the medium was measured by a spectrophotometric assay based on the Griess reaction, as described by our previous works^15,17^.

### Determination of intracellular oxygen free radicals

Reactive oxygen species (ROS) were determined by estimating the fluorescence intensity from cultures after loading with cell-permeable fluorogenic probes H2DCFDA, which detects ROS and assesses the degree of overall oxidative stress, and DHR-123, which detects peroxide and peroxynitrite. After finishing the indicated treatments, cultures were washed once and replaced by fresh PBS. Then cells were incubated with H2DCFDA (10 μM) or DHR-123 (20 μM) for 30 min at 37 °C. The fluorescence of H2DCFDA undergoing ROS oxidation was measured at λex = 485 nm and λem = 528 nm while the intensity of fluorescent DHR-123 oxidation product (rhodamine 123) was measured at λex = 488 nm and λem = 515 nm by using a fluorometric microplate reader (FLx800 Fluorescence Reader, BioTek Instruments, Inc.). All fluorescence intensity readings (relative fluorescence unit, RFU) were normalized to the background value before comparing the sample to the untreated control.

### Immunofluorescence staining of LC3B

Cells grown on glass coverslips were fixed with 4% paraformaldehyde, permeabilized, blocked with 3% BSA/2% goat serum, and incubated with the LC3B antibody at 4 °C overnight. After washes, cells were incubated with the corresponding secondary antibody conjugated with Alexa Fluor 568 in dark for 2 h and analyzed using a laser confocal microscope (MRC-1000; Bio-Rad, Hercules, CA; LSM510 META, Carl Zeiss, Oberkochen, Germany). 4′, 6-diamidino-2-phenylindole (DAPI) was used to label cell nucleus. To assess autophagy, the cells with punctate LC3B fluorescence were specially noted and total number of LC3B puncta per DAPI-labelled cell was counted. At least 200 cells were examined in each treatment group per experiment. A comparison of LC3B puncta formation per cell between the indicated groups was performed.

### Enzyme-linked immunosorbent assay (ELISA) for TNF-α

A commercial ELISA kit was applied to estimate the levels of mouse TNF-α released from cultured cells according to the manufacturer’s instructions.

### TUNEL assay

According to the manufacturer’s instructions, a TUNEL assay was applied to detect apoptosis. The TUNEL-positive cells were visualized under an inverted fluorescence microscope (Nikon ECLIPSE Ts2 microscope, Nikon, Inc.) equipped with a standard FITC filter and a digital camera (Diagnostic Instruments, Sterling Heights, MI, USA). For quantification, matching bright-field and fluorescent TUNEL-FITC photos were taken for each field. At least 25 fields were examined in each treatment group per experiment. The cell percentage showing fluorescent TUNEL-positive nuclei in a single field is determined by assuming a 100% of total cell number in the matching bright-field photo.

### Western blots

Cells were harvested in ice-cold RIPA buffer (50 mM Tris/HCl, pH 8.0, 150 mM NaCl, 1% Nonidet P-40, 0.5% sodium deoxycholate, 0.1% SDS) containing protease and phosphatase inhibitors (Gold Biotechnology Inc., St. Louis, MO, USA). Lysis occurred on ice for 20 min and then total lysates were cleared by centrifugation at 14,000 × g for 10 min (4 °C). A Bio-Rad protein assay was used for protein quantification (Bio-Rad, Hercules, CA, USA). Cell lysate (40 μg) was resolved by SDS–polyacrylamide gel electrophoresis and electroblotted onto polyvinylidene fluoride membranes (Millipore, Bedford, MA, USA). After blocking (5% non-fat dry skim milk), the membranes were immunoblotted for desired proteins. The immunoblots developed on X-ray film were visualized by an enhanced chemiluminescence system (Amersham Biosciences, Piscataway, NJ, USA). The intensities of the protein bands were quantified using Image J software (NIH, Bethesda, Maryland, USA).

### Statistical analysis

SigmaStat version 4.0 software from Jandel Scientific (San Diego, CA, USA) was applied. For experiments involving multiple treatment groups, statistical significant difference was analyzed using one-way ANOVA, followed by Dunnett’s test or Bonferroni’s t-test. For analysis between two groups, an independent sample t-test (2-tailed) was used to evaluate differences. All data are presented as mean ± standard error of the mean (SEM). A value was considered significant if the probability value was less than 0.05 (*p* < 0.05).

## Supplementary information

Supplementary Information

## Data Availability

All data generated or analyzed during this study are included in this published article and its Supplementary information files.
