# Supplementary material for: Acute glucose fluctuation impacts microglial activity, leading to inflammatory activation or self-degradation
Source: Sci Rep. 2019 Jan 29;9:840. doi: 10.1038/s41598-018-37215-0 (PMC6351546; doi:10.1038/s41598-018-37215-0)
Supplement: Supplementary file 1 — Supplementary Information [file 41598_2018_37215_MOESM1_ESM.pdf]

Supplementary information

Acute glucose fluctuation impacts microglial activity, leading to inflammatory activation or self-degradation

Cheng-Fang Hsieh [1, 2, 3, +], Ching-Kuan Liu [1, 2, +], Ching-Tien Lee [4], Liang-En Yu [1] & Jiz-Yuh Wang [1, 5, *]

[1] Graduate Institute of Medicine, College of Medicine, Kaohsiung Medical University, Kaohsiung, Taiwan.

[2] Department of Neurology, Kaohsiung Medical University Hospital, Kaohsiung Medical University, Kaohsiung, Taiwan.

[3] Division of Geriatrics and Gerontology, Department of Internal Medicine, Kaohsiung Medical University Hospital, Kaohsiung, Taiwan.

[4] Department of Nursing, Hsin-Sheng College of Medical Care and Management, Taoyuan, Taiwan.

[5] Department of Medical Research, Kaohsiung Medical University Hospital, Kaohsiung, Taiwan.

[+] Cheng-Fang Hsieh and Ching-Kuan Liu contributed equally to this work.

* Correspondence and requests for materials should be addressed to Jiz-Yuh Wang (jizyuhwang@cc.kmu.edu.tw)

Methods

Primary microglial cultures

Microglial cells were cultured according to a previously established protocol [1]. In brief, after sacrifice, the brain tissues of newborn Sprague Dawley (SD) rats were mechanically removed from the crania. After aseptic removal of the meninges and blood vessels, the brains were dissociated mechanically with scissors. Next, cell aggregations were suspended in 0.25% Trypsin-EDTA solution (10 min, 37℃) and gentle trituration was performed using a pipette. The digested cells were filtered through an 80-μm pore mesh, pelleted and suspended in 10% FBS/DMEM. The brain cells were cultured on poly-L-lysine pre-coated flasks, and the medium was frequently refreshed at an interval of 2 days until the cell composition contained < 5% neurons. Subsequently, the microglial cells were separated by gently shaking (180 rpm, 5 h, 37℃) the flasks after a confluent monolayer formed (10−14 days) and were reseeded into multi-well plates at a density of 2×10^5 cells/ml. These microglia were separately cultured in NG or HG medium and then used for the experiments within 36–48 h, at which point the cells were well attached. The purity was 95–98% as determined by immunocytochemistry for the microglia-specific marker, OX-42.

Cell viability assay

Cell survival was evaluated by the VisionBlue™ Quick Cell Viability Fluorometric Assay Kit (BioVision, Mountain View, CA, USA) according to the manufacturer's instructions. This fluorescence-based single-step cell viability assay utilizes the redox dye (resazurin) which is not fluorescent, but upon reduction by metabolically active cells, it becomes highly fluorescent. Therefore, viable metabolically active cells can be measured. Initially, cells were seeded on 96-well culture plates at a density of 1×10^5 cells/ml in a volume of 200 μl of cell medium. After completing the desired treatments, fresh cell medium containing 1/10 volume of VisionBlue reagent was added to each well, followed by a 37°C incubation for 2 h. The fluorescence (excitation: 540 nm; emission: 590 nm) was measured using a fluorometric microplate reader (FLx800 Fluorescence Reader, BioTek Instruments, Inc.). All data were normalized to background values.

Discussion

Glucose is the major energy source for the brain. The glucose concentration in the brain is approximately 20% of that of plasma glucose concentration [2]. Five main glucose transporters (GLUT 1–5) mediate glucose uptake in the brain and various peripheral tissues [3]. Microglia can express various GLUTs under different circumstances, and previous studies have indicated different GLUTs to be expressed in microglia to ensure sufficient glucose influx to meet the energy demand. Given that GLUT1 and GLUT3 (a main neuronal GLUT) are expressed in many tissues at variable levels and are thought to regulate basal glucose uptake [4], and that GLUT4 is only expressed in discrete brain areas [5], the protein levels of GLUT1 were examined to ensure a constant and stable influx of glucose in both NG- and HG-cultured BV-2 cell lines even if glucose levels are low, and to further explore whether primitive GLUT1 expression is disturbed by glucose fluctuations in this study. Regarding the plasma membrane glucosensors, neurons contain the Kir6.2 subunit of the ATP-dependent potassium channel (K-$ATP_{Kir6.2}$), SGLTs [6], and GLUT2 [7-9]; additionally, glial cells express glucosensors, including the GLUT2 and Kir6.2 subunit of the K-ATP channel [7,8]. Although these brain cells also express other glucose transporters, they are currently not regarded as sensors. Interestingly, defects in hypothalamic and brainstem membrane glucosensors, in particular, GLUT2 and sweet taste receptors, have been implicated in some diseases involving disorders in brain glucose metabolism such as obesity, diabetes mellitus, multiple sclerosis, Alzheimer's, Parkinson's, and Huntington's diseases [10]. Therefore, in this study, GLUT2 was the first priority to be examined.

Interestingly, microglia appear to be the only cells in the CNS expressing GLUT5 [11]. The exact function of GLUT5 in microglia in relation to glucose metabolism is not known. GLUT5 has a low affinity for glucose, and its affinity for fructose is much higher. It has been proposed that microglial GLUT5 expression is not correlated with prolonged hyperglycemia and that GLUT5 expressed in the endothelial cells and microglia of the brain is responsible for the transport of fructose. Fructose feeding in both young and older adult rats increased the expression of both GLUT5 mRNA and protein in the brain [12]. It is plausible that increased brain expression of GLUT5 allows fructose to play a role as an alternative energy source, at least in the short term. Furthermore, fructose-fed rats have activated microglia and primary rat microglia cultured under high fructose conditions showed upregulation of inflammatory pathways [13]. Therefore, microglia may play a key role in fructose-induced metabolic disorders. Consistent with previous studies, although GLUT5 is regarded as a fructose transporter, we still examined it and indeed observed that the GLUT5 protein levels were not markedly affected in cultured BV-2 cells exposed to glucose fluctuations (Supplementary Fig. 2).

Recently, a study proposed by Zhang et al. indicated that an HG (35 mM) condition can augment LPS-induced rat microglial activation and inflammatory cytokine levels [14]. Despite some consistency with our results, an obvious dissimilarity to the present study is that Zhang et al. did not remark on the viewpoint related to any GLUTs. Additionally, the brain glucose

concentration is thought to be 5-fold lower than that of plasma (20–30 mM in diabetic mice) [2]; yet, microglia were illogically cultured *in vitro* under 25–50 mM glucose conditions, a concentration range rarely observed in the brain during physiological or pathophysiological conditions. Therefore, compared to the study by Zhang et al., we think that using a glucose fluctuation between 5.5 mM (NG) and 25 mM (HG) in the present study may be more appropriate to offer new insight into the pathophysiology of diabetic neurodegeneration.

References

1 Wang, J. Y., Lee, C. T. & Wang, J. Y. Nitric oxide plays a dual role in the oxidative injury of cultured rat microglia but not astroglia. *Neuroscience* **281**, 164-177, doi:10.1016/j.neuroscience.2014.09.048 (2014).

2 Dunn-Meynell, A. A. *et al.* Relationship among brain and blood glucose levels and spontaneous and glucoprivic feeding. *J Neurosci* **29**, 7015-7022, doi:10.1523/JNEUROSCI.0334-09.2009 (2009).

3 Thorens, B. & Mueckler, M. Glucose transporters in the 21st Century. *Am J Physiol Endocrinol Metab* **298**, E141-145, doi:10.1152/ajpendo.00712.2009 (2010).

4 Kalsbeek, M. J., Mulder, L. & Yi, C. X. Microglia energy metabolism in metabolic disorder. *Mol Cell Endocrinol* **438**, 27-35, doi:10.1016/j.mce.2016.09.028 (2016).

5 Leloup, C. *et al.* Discrete brain areas express the insulin-responsive glucose transporter GLUT4. *Brain Res Mol Brain Res* **38**, 45-53 (1996).

6 Kang, L., Routh, V. H., Kuzhikandathil, E. V., Gaspers, L. D. & Levin, B. E. Physiological and molecular characteristics of rat hypothalamic ventromedial nucleus glucosensing neurons. *Diabetes* **53**, 549-559 (2004).

7 Shah, K., Desilva, S. & Abbruscato, T. The role of glucose transporters in brain disease: diabetes and Alzheimer's Disease. *Int J Mol Sci* **13**, 12629-12655, doi:10.3390/ijms131012629 (2012).

8 Garcia, M. *et al.* Hypothalamic ependymal-glial cells express the glucose transporter GLUT2, a protein involved in glucose sensing. *J Neurochem* **86**, 709-724 (2003).

9 Koekkoek, L. L., Mul, J. D. & la Fleur, S. E. Glucose-Sensing in the Reward System. *Front Neurosci* **11**, 716, doi:10.3389/fnins.2017.00716 (2017).

10 Welcome, M. O. & Mastorakis, N. E. Emerging Concepts in Brain Glucose Metabolic Functions: From Glucose Sensing to How the Sweet Taste of Glucose Regulates Its Own Metabolism in Astrocytes and Neurons. *Neuromolecular Med* **20**, 281-300, doi:10.1007/s12017-018-8503-0 (2018).

11 Horikoshi, Y. *et al.* Human GLUT5 immunolabeling is useful for evaluating microglial status in neuropathological study using paraffin sections. *Acta Neuropathol* **105**, 157-162, doi:10.1007/s00401-002-0627-4 (2003).

12 Shu, H. J., Isenberg, K., Cormier, R. J., Benz, A. & Zorumski, C. F. Expression of fructose sensitive glucose transporter in the brains of fructose-fed rats. *Neuroscience* **140**, 889-895, doi:10.1016/j.neuroscience.2006.02.071 (2006).

13 Li, J. M. *et al.* Betaine recovers hypothalamic neural injury by inhibiting astrogliosis and inflammation in fructose-fed rats. *Mol Nutr Food Res* **59**, 189-202, doi:10.1002/mnfr.201400307 (2015).

14 Zhang, X. *et al.* Enhancement of LPS-induced microglial inflammation response via TLR4 under high glucose conditions. *Cell Physiol Biochem* **35**, 1571-1581, doi:10.1159/000373972 (2015).

Supplementary Fig. 1

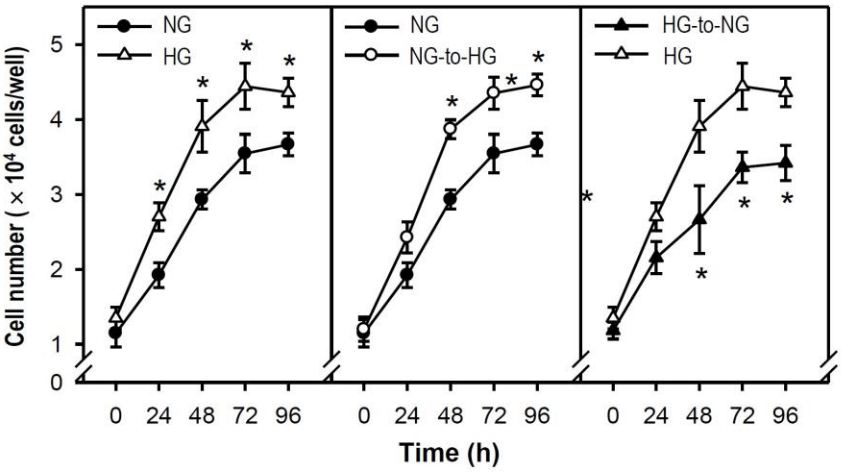

Supplementary Figure 1. The BV-2 cell proliferation in *in vitro* cultures is strengthened and weakened by the NG-to-HG and HG-to-NG shifts, respectively. The BV-2 cells were constantly cultured in NG and HG media, separately (i.e., NG-cultured cells and HG-cultured cells, respectively), and then treated with or without a glucose shift (i.e., NG, NG-to-HG, HG, and HG-to-NG) at an indicated time point of 0 h. Four groups of cultures were continuously incubated for different times ranging from 0 to 96 h. At the specified time, cells were trypsinized, resuspended and counted with a Cellometer Auto T4 instrument. Each point represents the mean ± SEM from four independent experiments performed in triplicate. *p < 0.05 versus constant NG group (left and middle panels) or constant HG group (right panel) at the same time point.

Supplementary Fig. 2

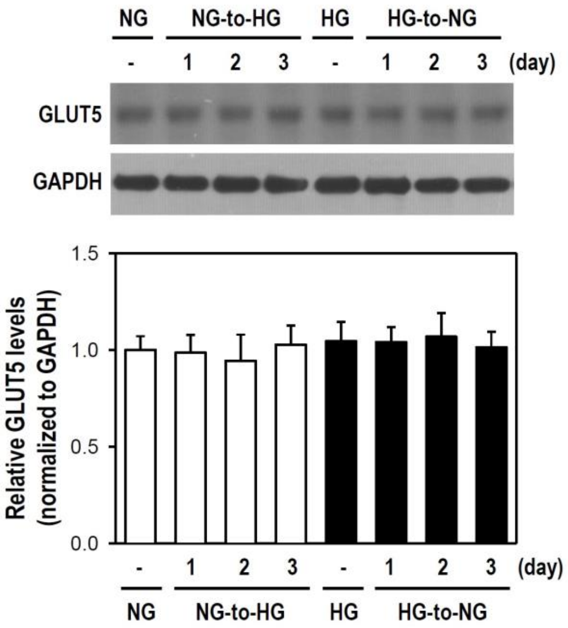

Supplementary Figure 2. Neither NG-to-HG nor HG-to-NG shift impacts on the expression level of GLUT5 in cultured BV-2 microglia. The media used to maintain NG- and HG-cultured cells were replaced by HG and NG media, respectively, and then cultures were consecutively incubated for 1, 2, or 3 days. After harvest, cells were subjected to western blotting for the determination of GLUT5 expression. The representative blots are presented and GAPDH served as a protein loading control. The histogram illustrates relative levels obtained by quantifying the western blot band intensity. The corresponding GAPDH levels were used for normalization, and the NG-cultured cell group was assigned a value of 1. Data are represented as mean ± SEM from four independent experiments.

Supplementary Fig. 3

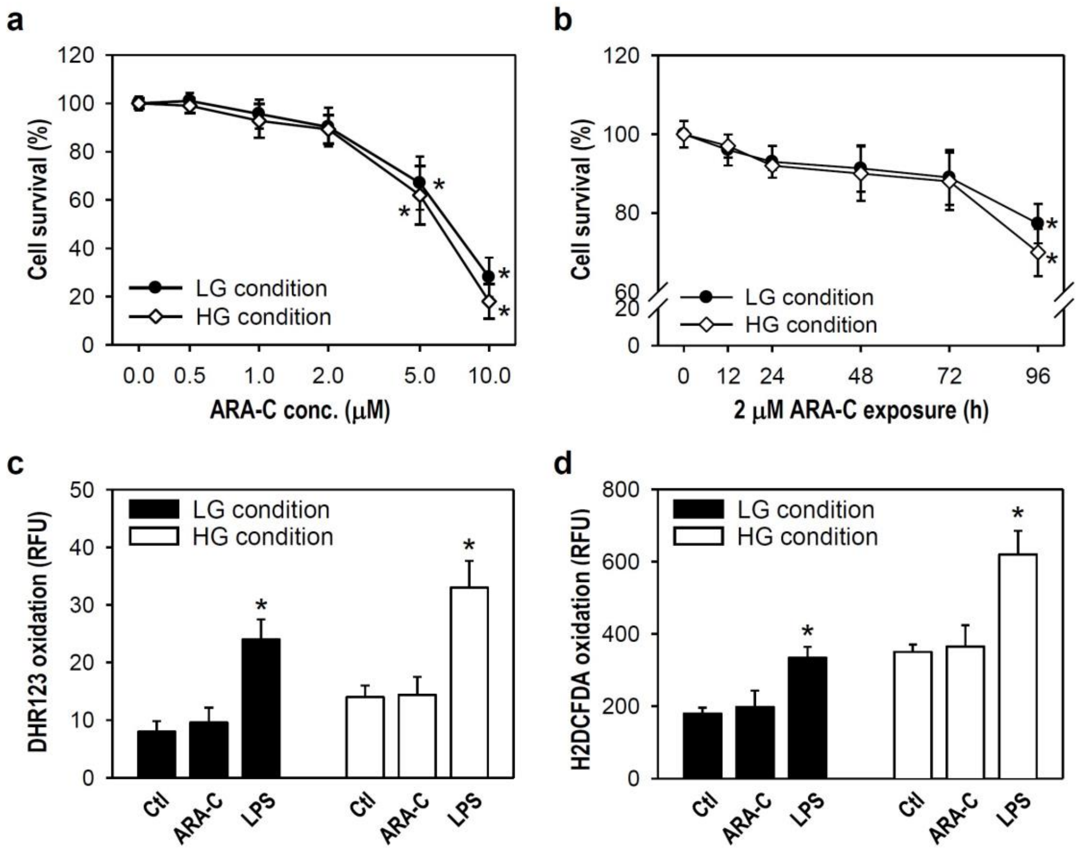

Supplementary Figure 3. ARA-C at concentrations of ≦ 2 μM is not a threat to cell viability and leads to no significant production of oxygen free radicals in cultured BV-2 microglia. (**a**) Concentration-response relationships were established after an exposure of cultures to various concentrations of ARA-C (0 to 10 μM, 24 h) in NG or HG condition. (**b**) Time course was determined after the treatment of cultures with 2 μM of ARA-C for different times (0 to 96 h) in NG or HG condition. The cell viability assay was used to determine BV-2 cell survival. The untreated cells (0 μM) in panel a and ARA-C-treated cells at 0 h in panel b served as the control. The number of live cells per well was calculated as a percentage of untreated control cell number. Each point represents mean ± SEM from at least three individual experiments performed in triplicate. *$p < 0.05$ versus untreated control group in the same glucose condition (**a**) or ARA-C-treated group at time point of 0 h in the same glucose condition. (**c,d**) As indicated, cells were treated with ARA-C (2 μM) or LPS (1 μg/ml) for 24 h in NG or HG condition. The untreated cells served as the control (Ctl). After harvest, cultures were subjected to the measurements of oxygen free radicals. Two fluorogenic probes, DHR-123 and H2DCFDA, were respectively employed to detect the intracellular production of ROS, especially peroxide and peroxynitrite (**c**), and the degree of overall oxidative stress (**d**). Data are represented as mean ± SEM from at least three individual experiments

performed in triplicate. *$p < 0.05$ versus untreated control group in the same glucose condition. RFU: relative fluorescence unit.

Supplementary Fig. 4

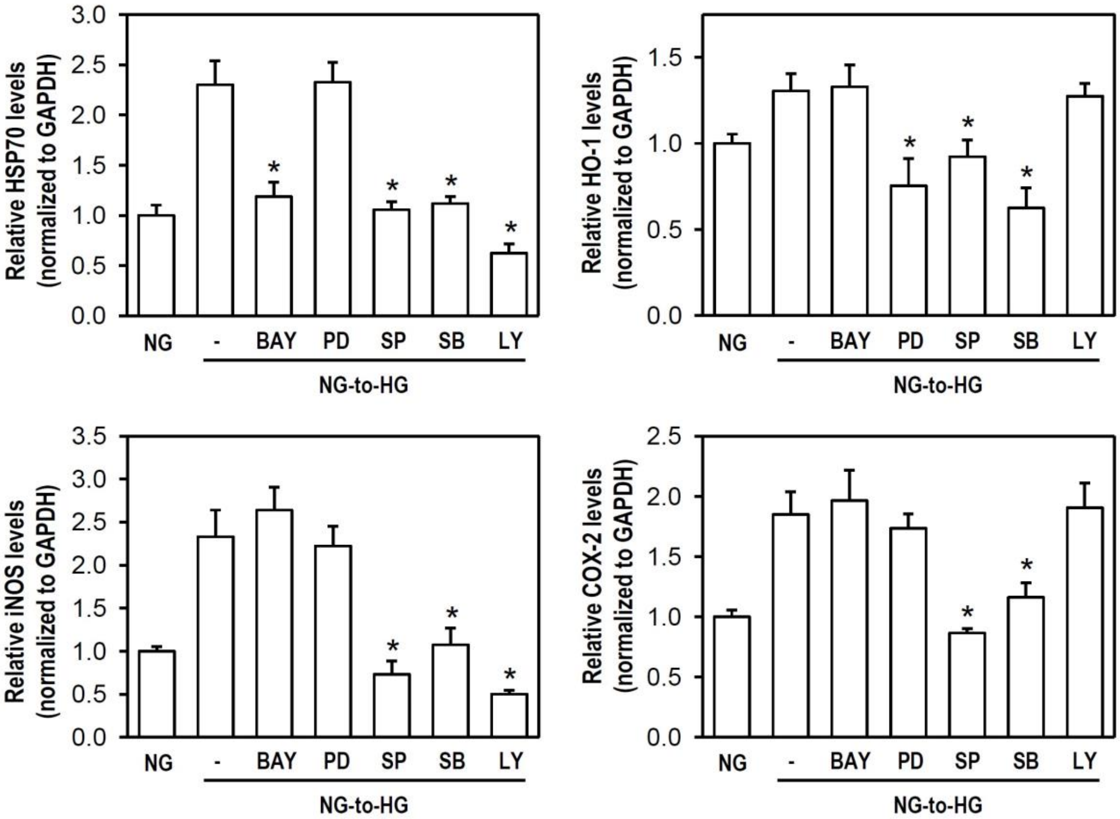

Supplementary Figure 4. The quantification was performed on the basis of the HSP70, HO-1, iNOS, and COX-2 expression levels in Figure 6a. The histograms show the relative levels obtained using densitometry to quantify western blot band intensity of each protein, followed by normalization to the corresponding loading control protein (GAPDH). Each quantitative value was expressed relative to the level of constant NG group (assigned a value of 1). Data are represented as the mean ± SEM from four independent experiments. *$p < 0.05$ versus NG-to-HG group treated with vehicle.

Supplementary Fig. 5

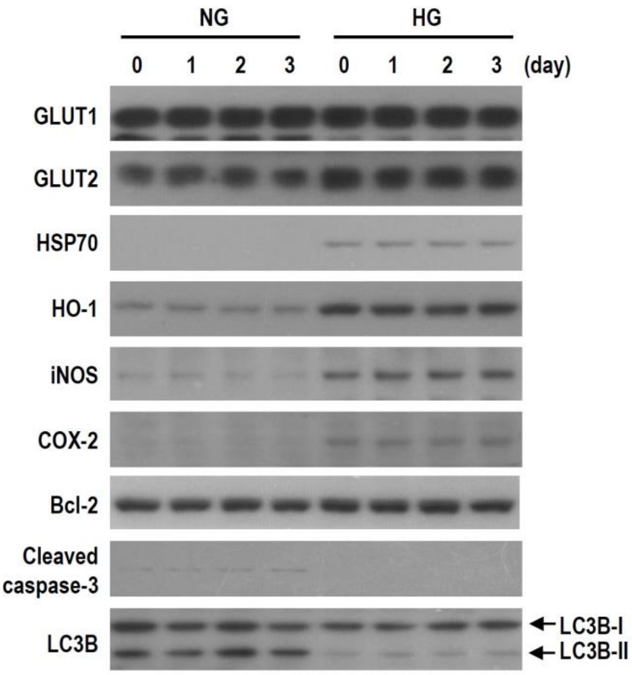

Supplementary Figure 5. A simple medium renewal causes no detectable marked alterations in protein expression levels from day 0 to 3 in NG- or HG-cultured BV-2 cells. The NG and HG media respectively used to maintain NG- and HG-cultured BV-2 cells were simply renewed on day 0 and then two BV-2 cell lines were consecutively incubated for 1, 2, or 3 days. After harvest, western blotting was performed to detect GLUT1, GLUT2, HSP70, HO-1, iNOS, COX-2, Bcl-2, cleaved caspase-3, and LC3B expression levels. The representative blots from one of three individual experiments are presented.

Supplementary Fig. 6

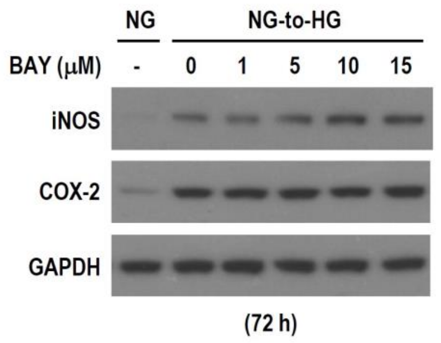

Supplementary Figure 6. BAY11-7082 shows no inhibitory effect on the inducible expression of iNOS and COX-2 after the exposure of BV-2 microglia to an NG-to-HG shift. NG-cultured cells were treated with different concentrations of BAY (0–15 μM) for 30 min before media being replaced from NG to HG. Cultures were continuously incubated in HG medium containing BAY at indicated concentrations for another 72 h. Control group was the NG-cultured cells treated with vehicle under a constant NG condition. After harvest, western blotting was used to detect the iNOS and COX-2 levels. GAPDH served as a protein loading control.

Supplementary Fig. 7

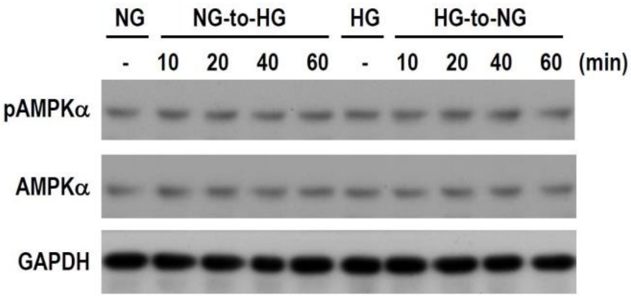

Supplementary Figure 7. No AMPK activation occurs in BV-2 microglia undergoing the shift of NG-to-HG or HG-to-NG. The media of NG- and HG-cultured BV-2 cells were simply renewed or replaced by HG and NG media, as indicated. Next, cultures were incubated for the time periods of 10, 20, 40, or 60 min. After harvest, western blotting was performed to detect the levels of pAMPKα and AMPKα. GAPDH served as a protein loading control.

Supplementary Fig. 8

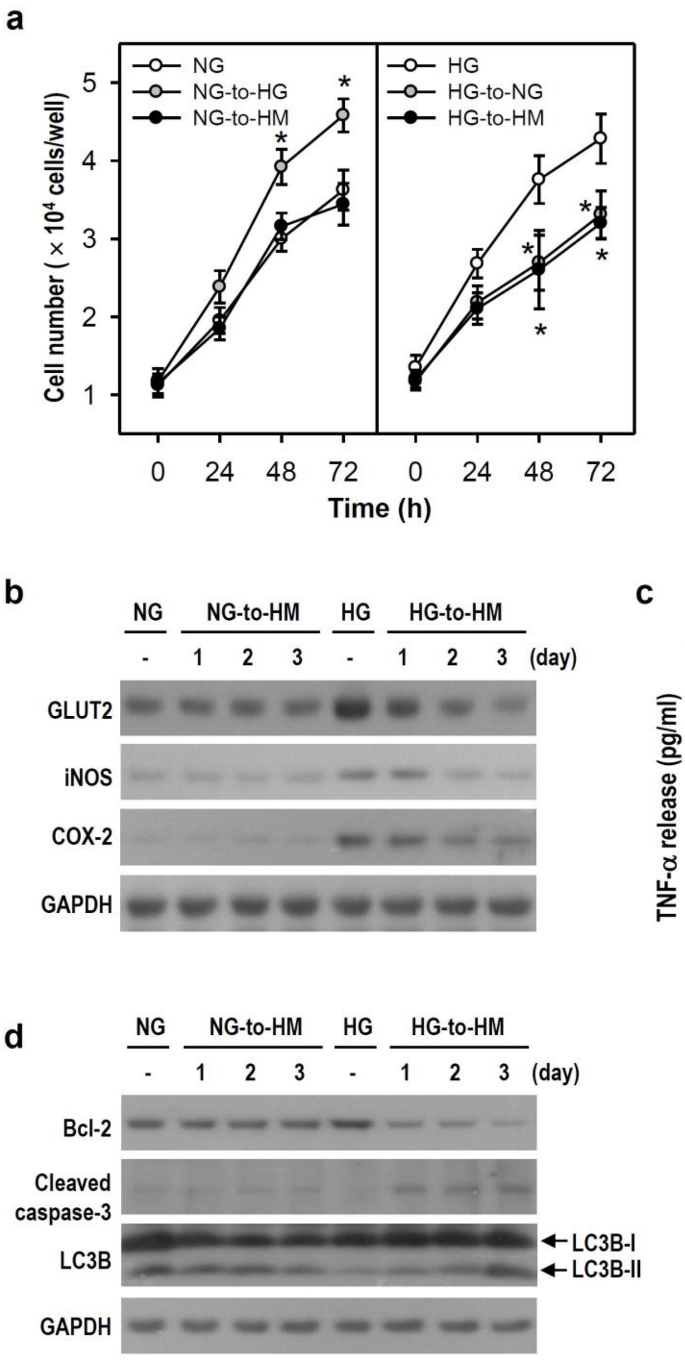

Supplementary Figure 8. High mannitol-induced hyperosmolarity exerts no effect on cell proliferation, inflammatory activation and inactive self-degradation in cultured BV-2 microglia. (**a**) Two BV-2 cell lines were separately cultured in NG and HG media (i.e., constant NG or HG). As indicated, some cells were exposed to a glucose shift (i.e., NG-to-HG or HG-to-NG) or treated with the high mannitol (i.e., NG-to-HM or HG-to-HM) for 0–72 h. After harvest, cells were subjected to a cell proliferation assay with the assistance of a

Cellometer Auto T4 instrument. Each point represents the mean ± SEM from at least three independent experiments performed in triplicate. *$p < 0.05$ versus constant NG group (left panel) or constant HG group (right panel) at the same time point. (**b**) As indicated, the media of both NG- and HG-cultured BV-2 cells were simply renewed or replaced by HM medium. After incubation for the indicated time points, western blotting was used to detect GLUT2, iNOS, and COX-2 levels. GAPDH served as a protein loading control. The representative blots from one of four independent experiments are presented. (**c**) The media of both NG- and HG-cultured BV-2 cells were simply renewed or replaced by NG, HG, and HM media, as indicated. After 48 h-incubation, an ELISA was applied to measure TNF-α releasing level. ARA-C (2 μg/ml) was added to cultures to eliminate the interfere of different proliferation rates. Data are represented as mean ± SEM from at least three individual experiments. *$p < 0.05$ versus constant NG group; $^{\#}p < 0.05$ versus constant HG group. (**d**) Similar to the treatments in panel b, western blotting was used to detect Bcl-2, cleaved caspase-3, and LC3B levels. GAPDH served as a protein loading control. The representative blots from one of four individual experiments are presented.

Supplementary Fig. 9

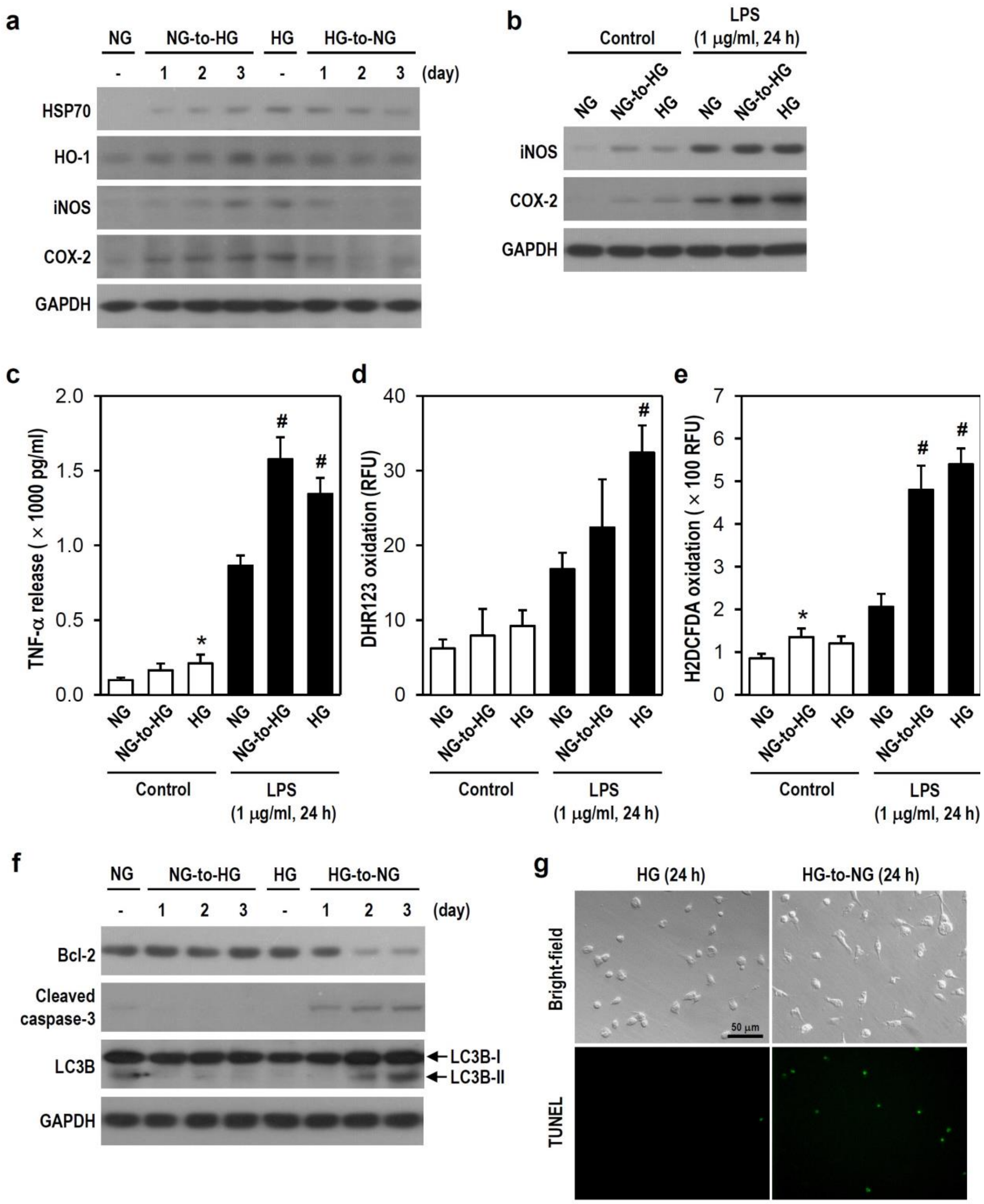

Supplementary Figure 9. Acute fluctuations of NG-to-HG and HG-to-NG separately induce inflammatory activation and inactive self-degradation in primary rat microglial cultures. (**a**) The microglial cell media appointed as NG and HG were simply renewed or replaced respectively by HG and NG media, as indicated. Next, cultures were consecutively incubated for 1, 2, or 3 days. After harvest, western blotting was performed to detect HSP70, HO-1,

iNOS, and COX-2 expression. GAPDH served as a protein loading control. The representative blots from one of four independent experiments are presented. (**b–e**) As indicated, both NG- and HG-cultured microglia were simply treated with or without LPS (1 μg/ml, 24 h); also, an NG-to-HG shift with or without LPS treatment was performed in NG-cultured microglia. After harvest, cultures were subjected to various biochemical assays for determining iNOS and COX-2 levels (**b**), TNF-α release (**c**), and productions of peroxides (**d**) and ROS (**e**). In panel b, GAPDH served as a protein loading control and the representative blots from one of four independent experiments are presented. Data are represented as mean ± SEM from four independent experiments performed in triplicate. $^{*}p < 0.05$ versus constant NG group; $^{\#}p < 0.05$ versus constant NG group treated with LPS. (**f**) Similar to the treatments in panel a, western blotting was executed to detect Bcl-2, cleaved caspase-3, and LC3B levels at the indicated time points. GAPDH served as a protein loading control. The representative blots from one of four individual experiments are presented. (**g**) A TUNEL assay was performed 24 h after the HG-to-NG shift or simple medium renewal. The representative photos of bright-field and matching TUNEL fluorescence are shown.

Supplementary Fig. 10

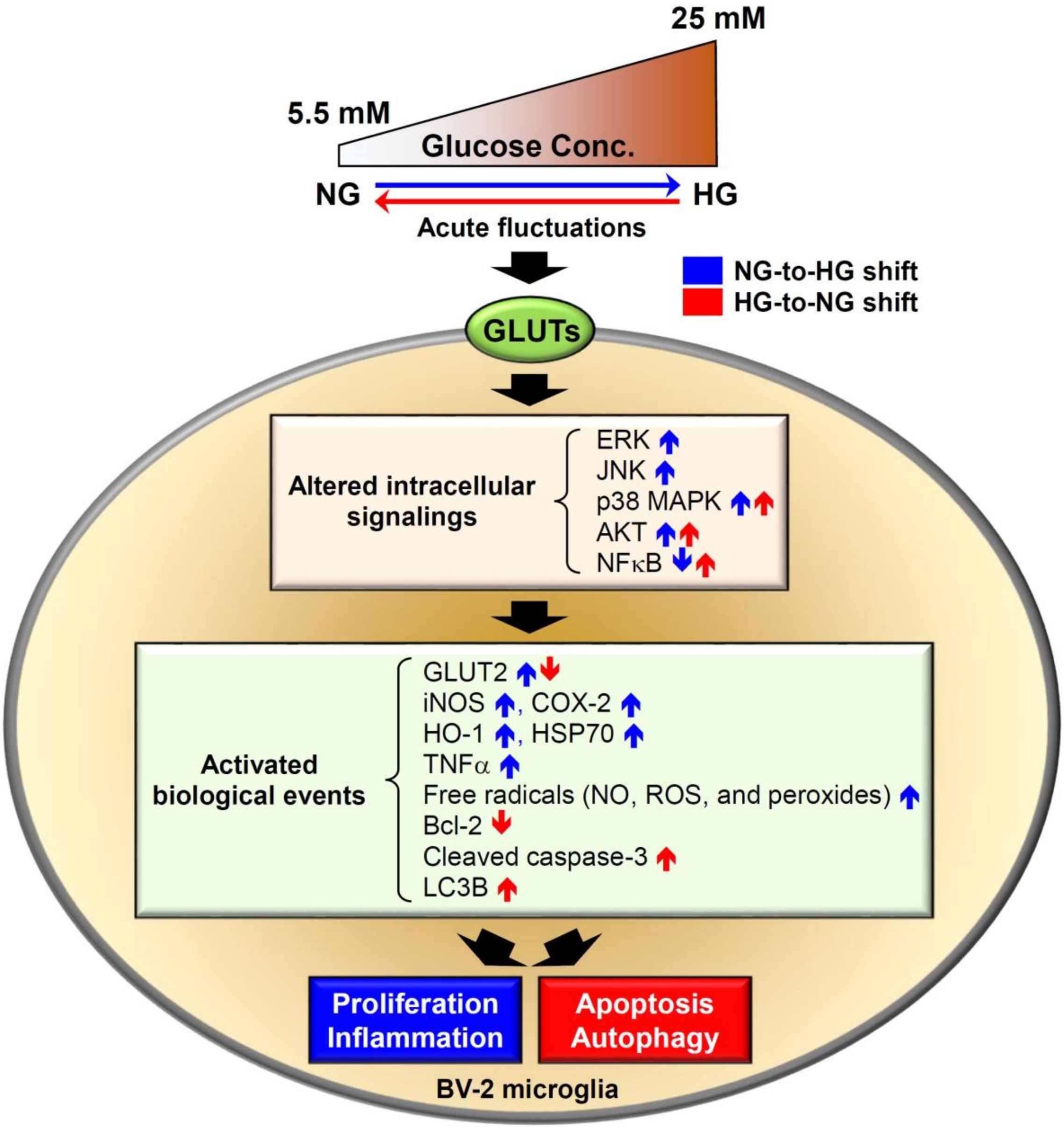

Supplementary Figure 10. A summary diagram shows the altered intracellular signalings and activated biological events in microglia, eventually leading to respective cell fates in response to the concentration shift of either NG-to-HG or HG-to-NG.

Original and uncropped images of the blots shown in Figure 1.

GLUT1 →
(M.W. 55 kDa)

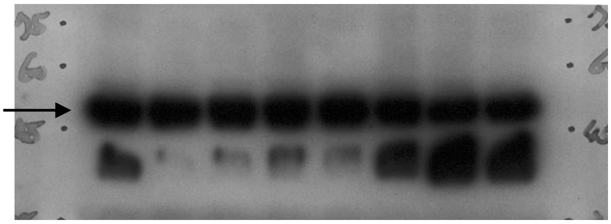

GLUT2
(M.W. 60-62 kDa)

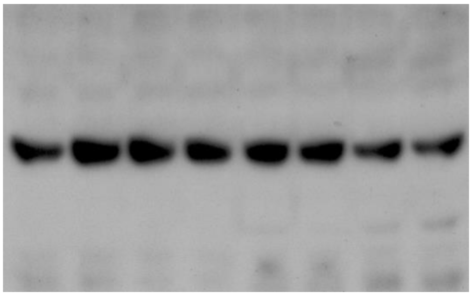

Immunoblot images for GLUT1 and GLUT2 shown in Fig. 1b

GLUT2
(M.W. 60-62 kDa)

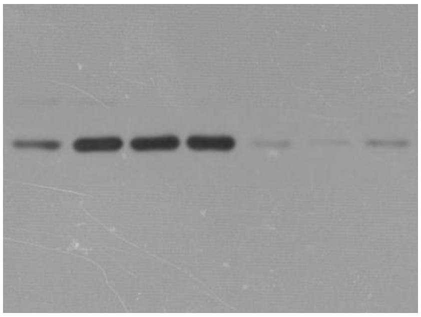

Immunoblot image for GLUT2 shown in Fig. 1d

Original and uncropped images of the blots shown in Figure 2.

HSP70
(M.W. 72, 73 kDa)

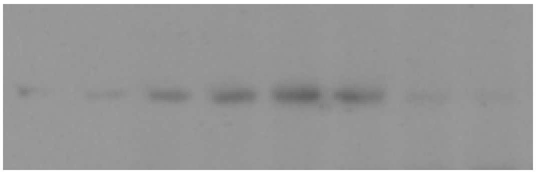

Immunoblot image for HSP70 shown in Fig. 2a

HO-1
(M.W. 32 kDa)

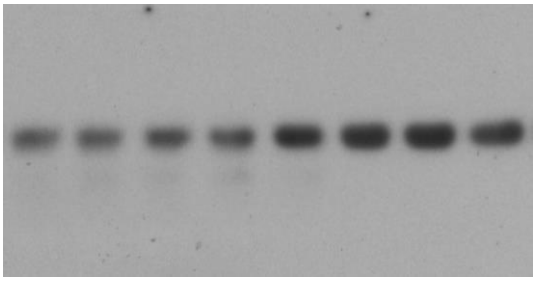

Immunoblot image for HO-1 shown in Fig. 2b

iNOS
(M.W. 130 kDa)

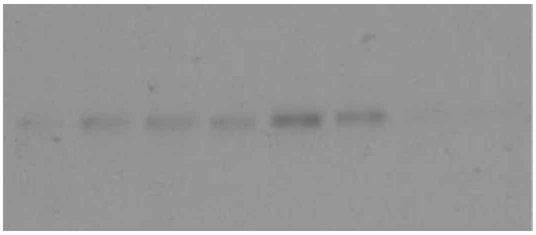

Immunoblot image for iNOS shown in Fig. 2c

COX-2
(M.W. 74 kDa)

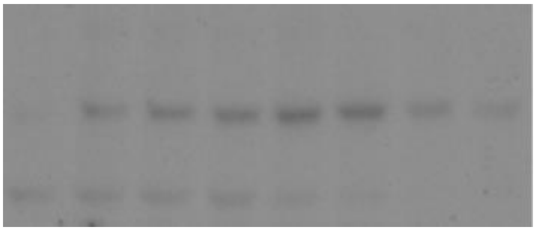

Immunoblot image for COX-2 shown in Fig. 2d

Original and uncropped images of the blots shown in Figure 4.

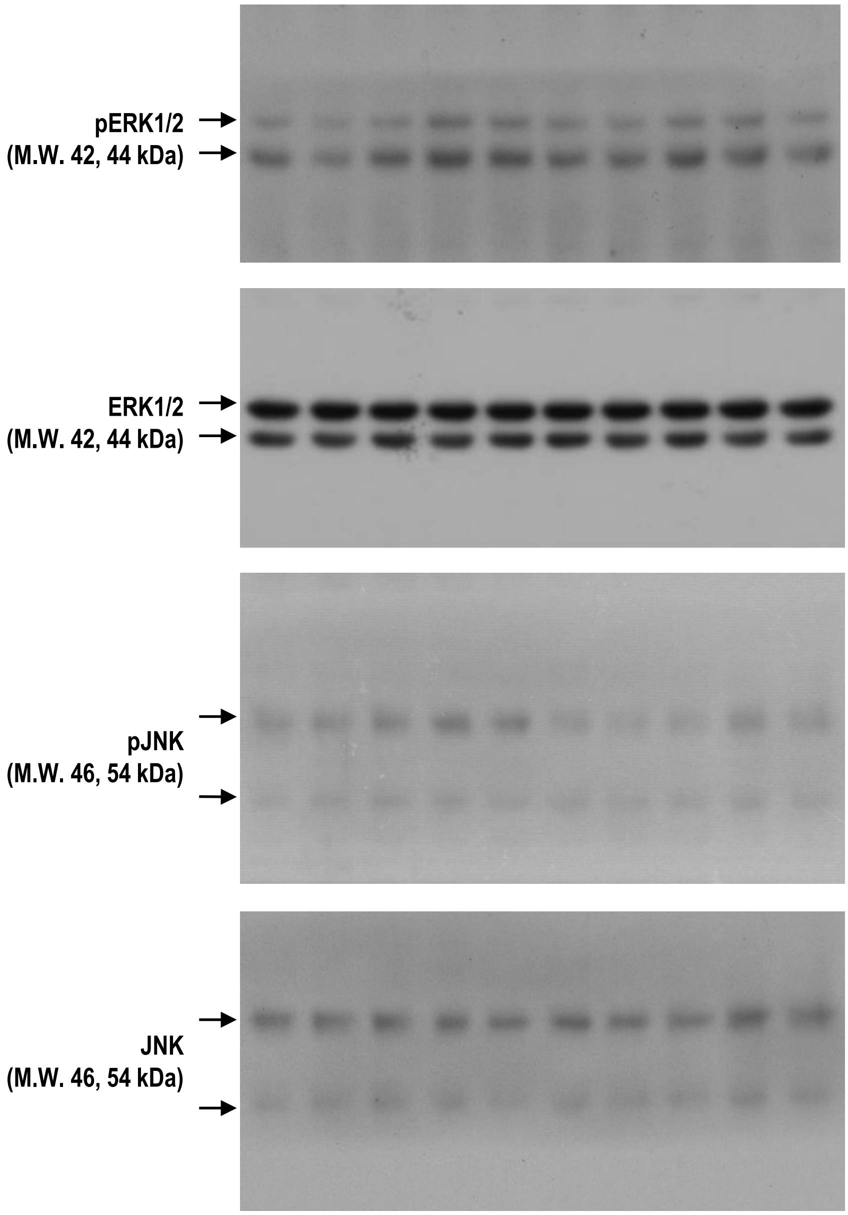

Immunoblot images for pERK, ERK, pJNK, and JNK shown in Fig. 4a

Original and uncropped images of the blots shown in Figure 4.

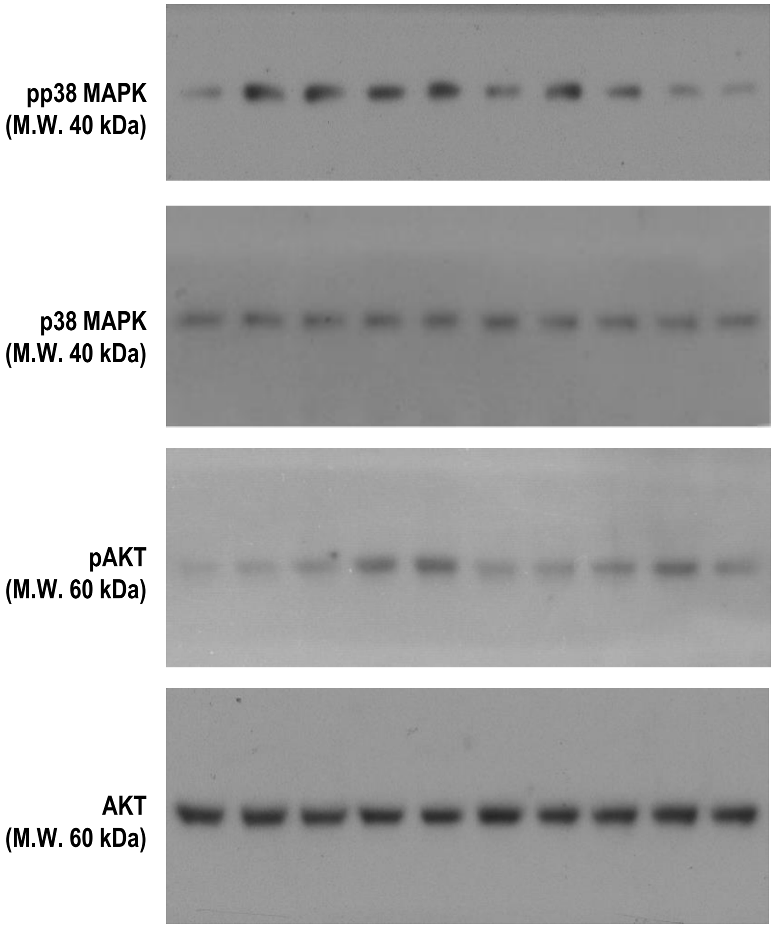

Immunoblot images for pp38 MAPK, p38 MAPK, pAKT, and AKT shown in Fig. 4a

Original and uncropped images of the blots shown in Figure 5.

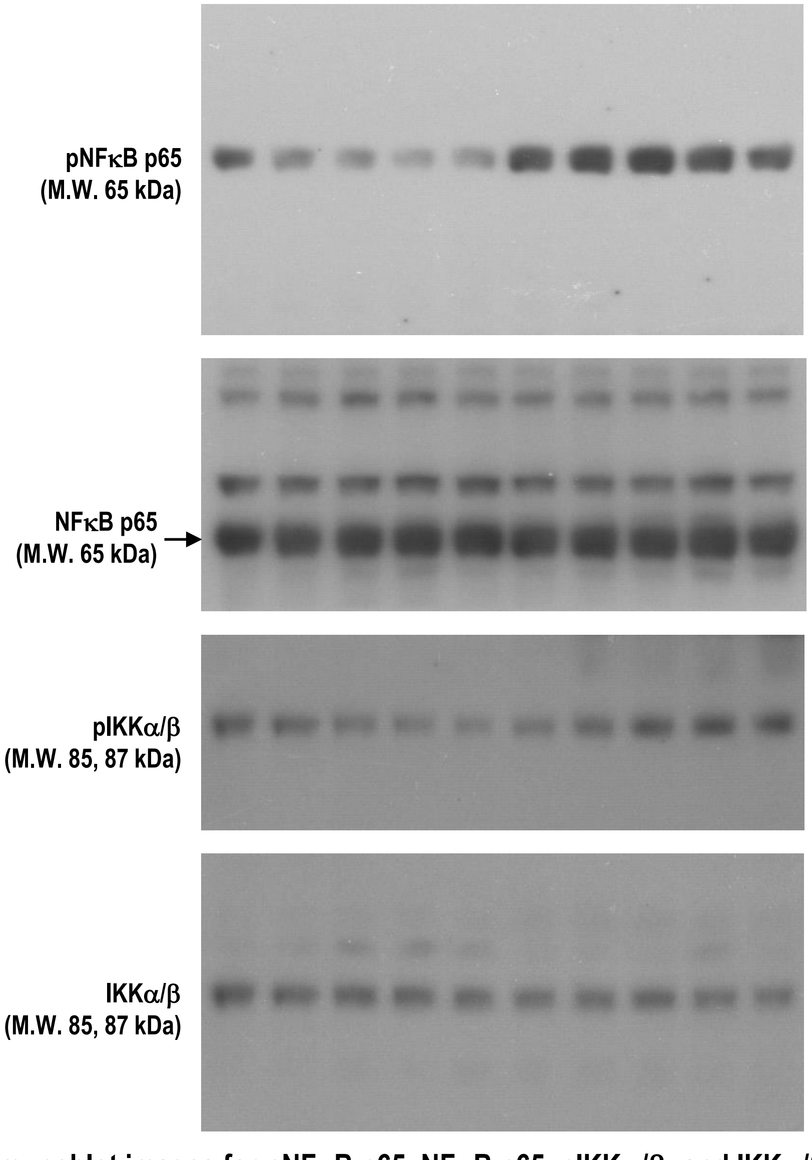

Immunoblot images for pNFκB p65, NFκB p65, pIKKα/β, and IKKα/β shown in Fig. 5a

Original and uncropped images of the blots shown in Figure 5.

pIκBα (M.W. 40 kDa)

IκBα (M.W. 40 kDa)

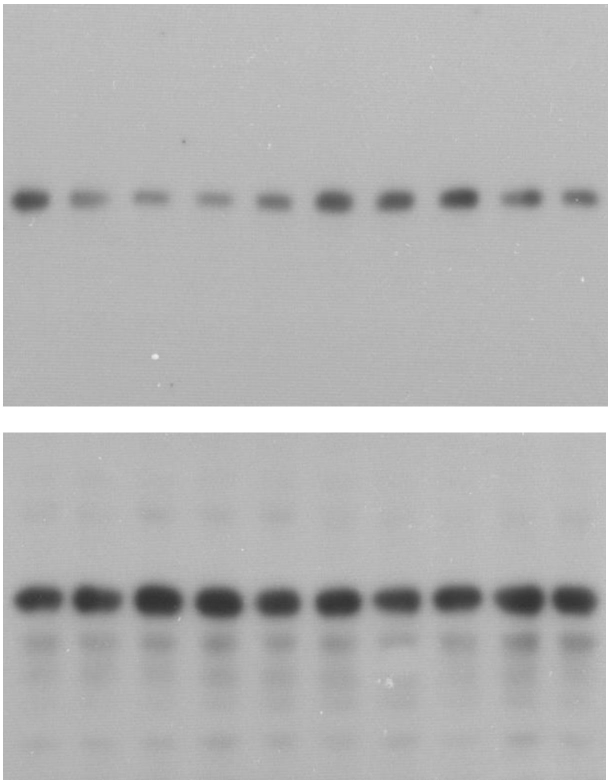

Immunoblot images for pIκBα and IκBα shown in Fig. 5a

Original and uncropped images of the blots shown in Figure 6.

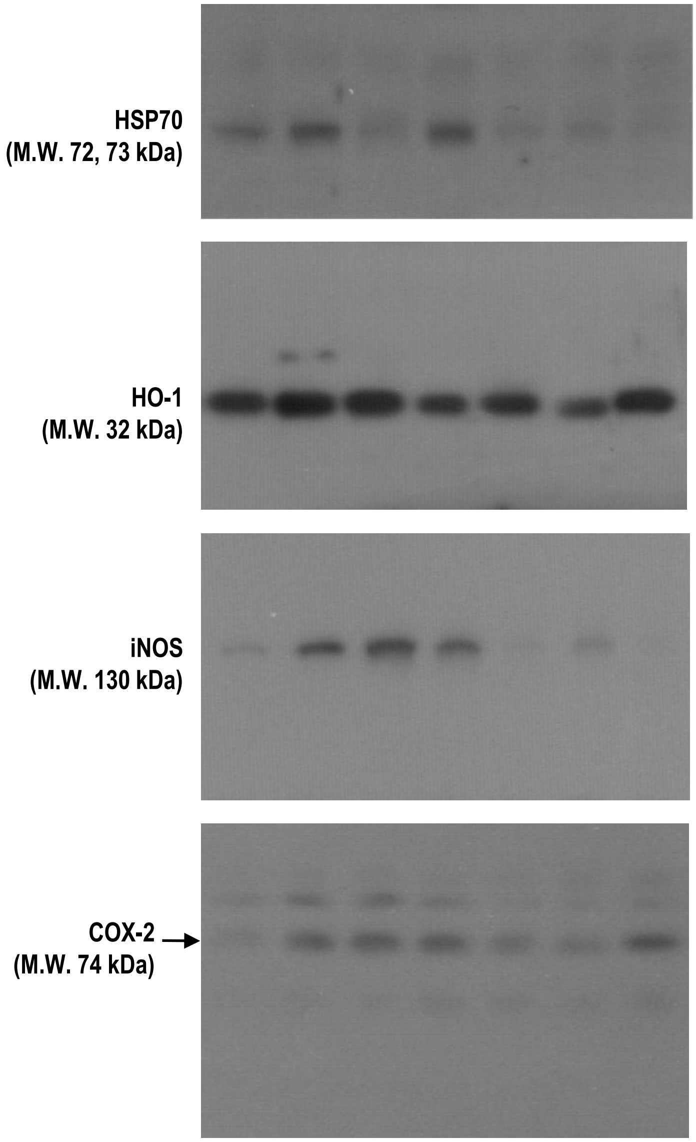

Immunoblot images for HSP70, HO-1, iNOS, and COX-2 shown in Fig. 6a

Original and uncropped images of the blots shown in Figure 7.

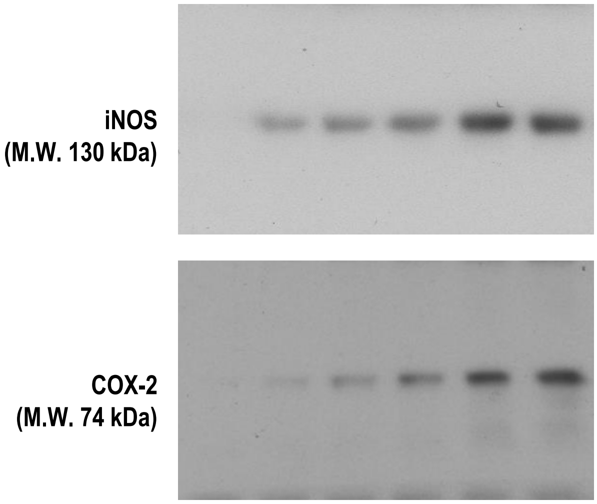

Immunoblot images for iNOS and COX-2 shown in Fig. 7a

Original and uncropped images of the blots shown in Figure 8.

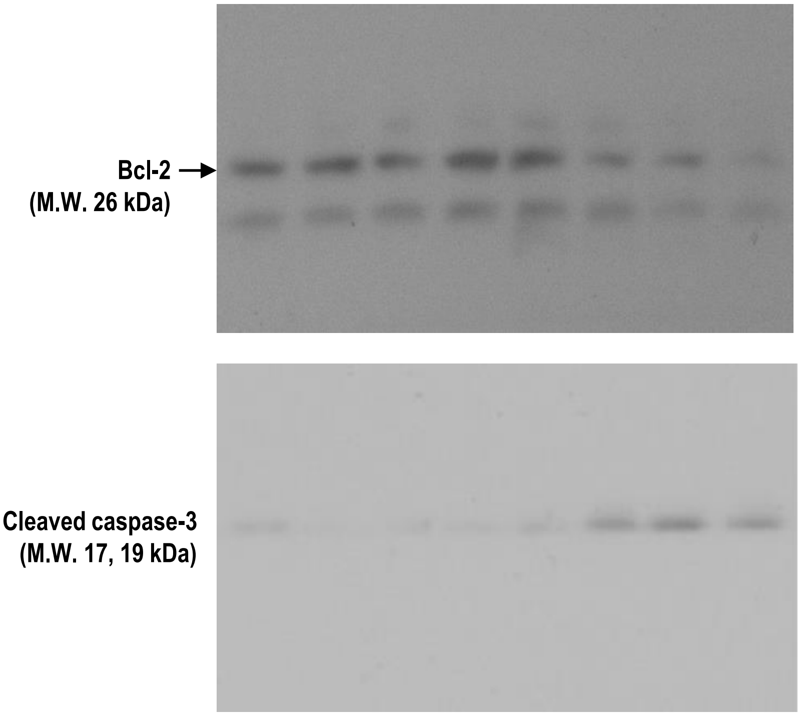

Immunoblot images for Bcl-2 and cleaved caspase-3 shown in Fig. 8a

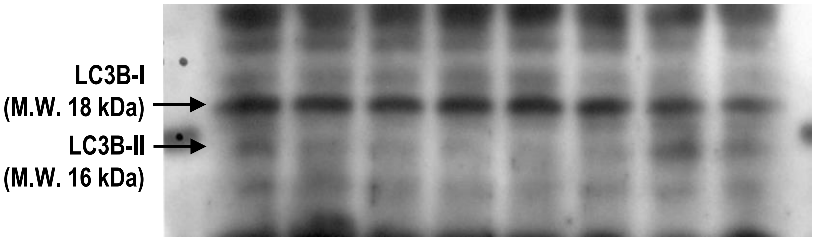

Immunoblot image for LC3B shown in Fig. 8d
